# Efficient Extraction of Polyphenols from *Hamamelis virginiana* Bark and Leaves: A Design-of-Experiments Approach to Accelerated Solvent- and Ultrasound-Assisted Extraction

**DOI:** 10.3390/molecules31213813

**Published:** 2026-10-22

**Authors:** Wiktoria Pacuła, Weronika Wójciak, Sławomir Dresler, Ireneusz Sowa, Agnieszka Skalska-Kamińska, Magdalena Wójciak

**Affiliations:** 1 Doctoral School, Medical University of Lublin, Aleje Raclawickie 1, 20-059 Lublin, Poland; weronikawojciak01@gmail.com; 2 Department of Analytical Chemistry, Medical University of Lublin, Aleje Raclawickie 1, 20-059 Lublin, Poland; slawomir.dresler@umlub.edu.pl (S.D.); ireneusz.sowa@umlub.edu.pl (I.S.); agnieszka.skalska-kaminska@umlub.edu.pl (A.S.-K.)

**Keywords:** witch hazel, antioxidant activity, polyphenols, flavonoids, ASE, UAE, response surface methodology

## Abstract

*Hamamelis virginiana* L. is a rich source of phenolic compounds with potential applications in pharmaceutical and cosmetic formulations. However, efficient recovery of these constituents depends strongly on the extraction technique and process conditions. The aim of this study was to optimize accelerated solvent extraction (ASE) and ultrasound-assisted extraction (UAE) of phenolic compounds from *H. virginiana* bark and leaves using a Design-of-Experiments (DoE) approach. The effects of solvent composition, extraction temperature, extraction technique, and number of extraction cycles were evaluated using total phenolic content (TPC), DPPH radical-scavenging activity, and selected phenolic constituents as response variables. The phytochemical profiles of the extracts were characterized by UHPLC-DAD-ESI/TOF-MS. The effects of the investigated extraction parameters differed among individual responses and between leaves and bark, with methanol concentration and the number of extraction cycles identified as particularly important factors. Multi-response optimization revealed matrix- and technique-dependent optimal extraction conditions. For leaves, the optimal conditions were 80 °C, 70.3% MeOH, and two cycles for ASE and 72 °C, 63.5% MeOH, and three cycles for UAE, whereas for bark they were 80 °C, 57.5% MeOH, and two cycles for ASE and 80 °C, 63.3% MeOH, and two cycles for UAE. Experimental validation showed generally good agreement between predicted and observed responses, with all but one experimental value falling within the corresponding 95% prediction intervals. These findings demonstrate that optimization of phenolic compound extraction from *H. virginiana* should account for both the plant matrix and the targeted chemical constituents and support the use of DoE as an effective approach for selecting ASE and UAE conditions.

## 1. Introduction

*Hamamelis virginiana* L. (witch hazel) is a medicinal plant widely used in pharmaceutical and cosmetic preparations, particularly those intended for the treatment of skin conditions involving inflammation, irritation, and oxidative stress [1,2]. The phytochemical profile of witch hazel comprises several classes of polyphenols, including hydrolysable tannins, flavonoids, phenolic acids, and proanthocyanidins, with their qualitative and quantitative distribution varying between different plant organs [3]. These compounds have attracted considerable interest due to their antioxidant, astringent, and anti-inflammatory activities and are regarded as major contributors to the biological effects of the plant [4,5]. Consequently, efficient extraction of the phenolic fraction is important for the development of polyphenol-rich *H. virginiana* preparations.

The recovery of phenolic compounds from plant materials is strongly influenced by the extraction technique and process variables, such as solvent composition, temperature, extraction time, and solvent-to-solid ratio [6]. Several solvent-based extraction techniques are commonly used for the recovery of phenolic compounds from *H. virginiana*, including maceration, ultrasound-assisted extraction (UAE), and heat reflux extraction (HRE) [7,8,9]. Recently, UAE, HRE, and ASE were compared for the extraction of polyphenols from *H. virginiana* leaves and bark, demonstrating a pronounced influence of the extraction approach on the recovery of individual phenolic constituents [3]. Unlike our previous study, which compared extraction techniques under predefined conditions, the present work applies a multivariable Design-of-Experiments (DoE) approach to evaluate the individual and interactive effects of extraction parameters, develop predictive response surface models, and identify matrix-specific optimal conditions through multi-response optimization.

Among the various extraction techniques, accelerated solvent extraction (ASE) and ultrasound-assisted extraction (UAE) are particularly attractive approaches for the efficient recovery of phenolic compounds from plant materials [10,11]. ASE employs solvents at elevated temperatures and pressures and is characterized by a high degree of automation and reproducibility [12]. In turn, UAE relies on acoustic cavitation, involving the formation and collapse of microscopic bubbles in the extraction medium, which facilitates disruption of the plant matrix [13]. Both techniques enhance solvent penetration, promote the release of intracellular constituents, and accelerate mass transfer. Extraction efficiency can be further enhanced by increasing the number of extraction cycles. However, intensive extraction conditions, including elevated temperatures or prolonged exposure to ultrasound, may adversely affect the stability of thermolabile constituents, while additional extraction cycles increase solvent consumption and processing time [14].

Both ASE and UAE are multivariable extraction processes whose efficiency and selectivity are determined by a combination of experimental conditions. Careful optimization of their operating parameters is therefore essential to maximize the recovery of target compounds while limiting their potential degradation and minimizing solvent consumption and processing time [10,15]. Conventional optimization based on a one-factor-at-a-time (OFAT) approach has important limitations, as it does not account for interactions between experimental factors [16]. In contrast, Design-of-Experiments (DoE) provides a systematic strategy for simultaneously evaluating the effects of multiple variables and their interactions while limiting the number of experimental runs [17,18].

Despite the considerable phytochemical and pharmacological interest in *H. virginiana*, systematic evaluation of the combined effects of ASE parameters on the recovery of phenolic constituents from its bark and leaves remains limited. In particular, the influence of the plant matrix on the relationships between extraction conditions, total phenolic recovery, antioxidant activity, and the extraction of individual phenolic compounds warrants further investigation. Therefore, the aim of the present study was to apply a Design-of-Experiments approach to investigate and optimize the extraction of phenolic compounds from *H. virginiana* bark and leaves using accelerated solvent extraction (ASE) and ultrasound-assisted extraction (UAE). The effects of extraction temperature, solvent composition, and the number of extraction cycles were systematically evaluated using total phenolic content, DPPH radical-scavenging activity, and the contents of individual phenolic compounds as analytical responses. The study further aimed to compare the performance of ASE and UAE and to determine whether the effects of extraction parameters were dependent on the plant matrix and the targeted phenolic constituents.

## 2. Results

### 2.1. Phytochemical Analysis

Prior to the optimization experiments, the phytochemical profiles of representative witch hazel extracts obtained by ultrasound-assisted extraction (UAE) and accelerated solvent extraction (ASE) were characterized using LC-MS to identify the major metabolites and select appropriate compounds for subsequent DoE-based optimization. No qualitative differences in the phytochemical profiles were observed between the two extraction techniques. The main identified metabolites are summarized in Supplementary Table S1, and representative chromatograms are presented in Supplementary Figure S1.

Overall, the identified constituents comprised galloyl derivatives and gallotannins, phenolic acids, catechin, and flavonol glycosides. Distinct differences were observed between the phytochemical profiles of witch hazel bark and leaves. Leaf extracts showed a more diverse phenolic profile, particularly due to the presence of numerous flavonol glycosides. Myricetin 3-O-rhamnoside and quercetin 3-O-rhamnoside were the predominant flavonoids, accounting for approximately 35.8–39.5% and 19.5–22.6%, respectively, of the total quantified flavonoid content (Supplementary Figure S2). In addition, leaves contained a greater number of highly galloylated, higher-molecular-weight tannins (*m*/*z* 939–1395). In contrast, bark extracts were characterized predominantly by lower-molecular-weight galloyl derivatives, including galloyl- and digalloyl-hexoses (Supplementary Figure S3).

For the DoE analysis, selected phenolic compounds were evaluated either individually or as grouped responses according to their structural similarity. Flavonoids were expressed as the sum of the quantified flavonoid compounds, whereas gallic acid and its derivatives, including galloylquinic acid isomers and di-O-galloylquinic acid, were expressed as a combined response. Hamamelitannin was evaluated separately due to its relevance as a characteristic constituent of *H. virginiana*. Due to the lack of authentic reference standards, high-molecular-weight galloyl-hexoses (tri-, tetra-, penta-, hexa-, hepta-, and octa-O-galloyl-hexoses) were not quantified individually; instead, the sum of their chromatographic peak areas was used as a response in the DoE analysis.

### 2.2. DoE-Based Optimization

The effects of extraction temperature (X1), methanol concentration (X2), extraction technique (X3; ASE or UAE), and number of extraction cycles (X4) on the extraction of the predominant phenolic constituents and antioxidant activity of *H. virginiana* leaves and bark were evaluated using a Design-of-Experiments (DoE) approach. Separate response surface models were developed for each analytical response and independently for leaf and bark samples. The fitted models were used to evaluate the effects of the investigated extraction parameters, including interaction and nonlinear effects. The complete experimental design matrices and the corresponding analytical responses for leaf and bark extracts are provided in Supplementary Tables S2 and S3, respectively. Detailed analysis of variance (ANOVA) results for the fitted response surface models are provided in the Supplementary Materials for leaf extracts (Table S4a–g) and bark extracts (Table S5a–f).

**Figure 1:**
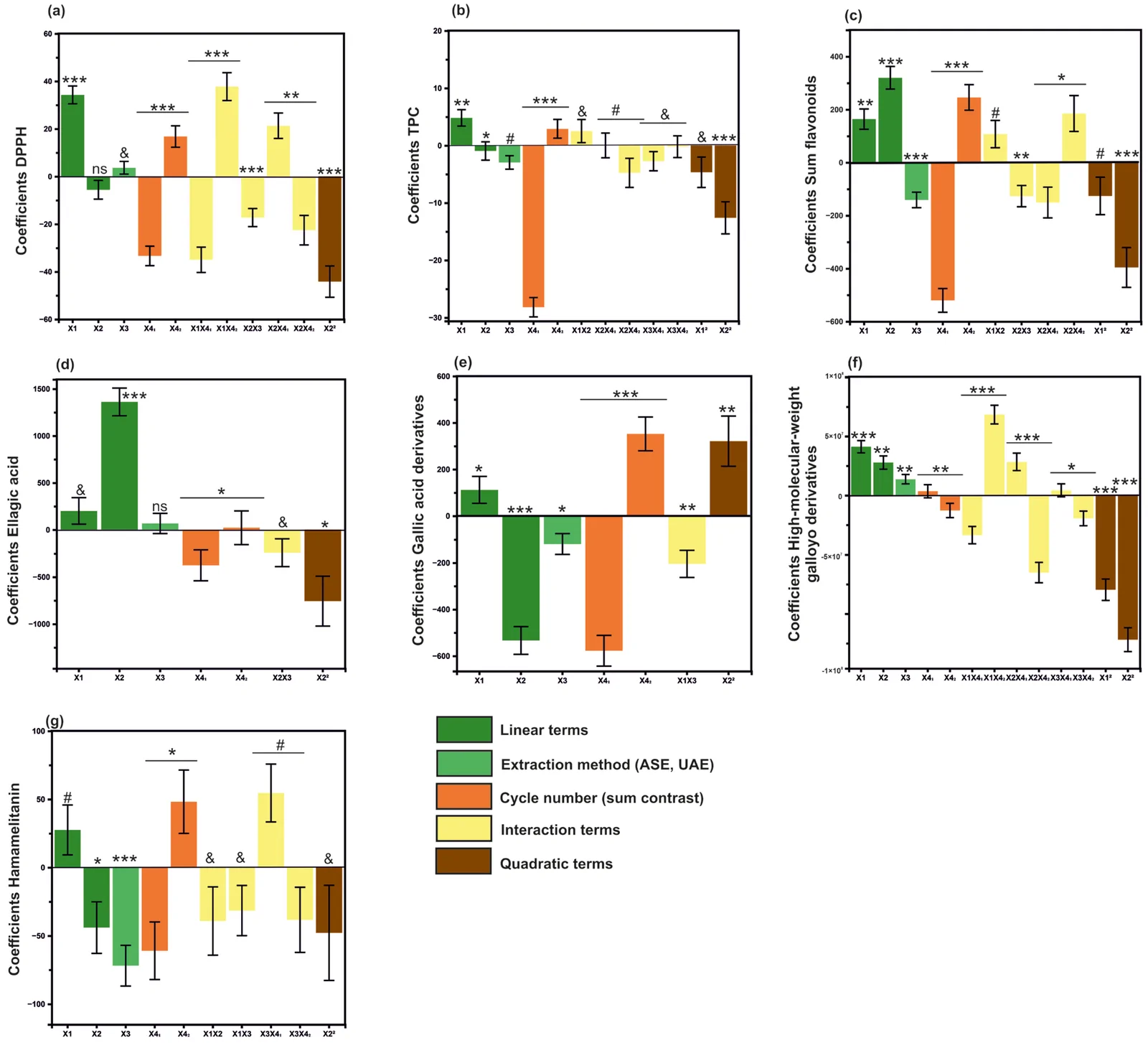
Regression coefficient plots of the significant effects of the fitted RSM models for leaf extracts: (**a**) DPPH radical-scavenging activity, (**b**) total phenolic content (TPC), (**c**) sum of flavonoids, (**d**) ellagic acid, (**e**) gallic acid derivatives, (**f**) high-molecular-weight galloyl derivatives, and (**g**) hamamelitannin. X1—extraction temperature, X2—methanol concentration (%), X3—extraction technique (ASE, UAE), X4—number of extraction cycles. Dark green bars represent linear terms, light green bars indicate the extraction method effect, orange bars correspond to the cycle number effect (sum contrasts), yellow bars denote interaction terms, and brown bars represent quadratic terms. Error bars indicate the uncertainty of the estimated regression coefficients. ns—*p* ≥ 0.15; & *p* < 0.15; # *p* < 0.10; * *p* < 0.05; ** *p* < 0.01; *** *p* < 0.001. The symbols indicate different *p*-value ranges for descriptive purposes only. Statistical significance was assessed at *p* < 0.05, and terms exceeding this threshold were not interpreted as statistically significant.

**Figure 2:**
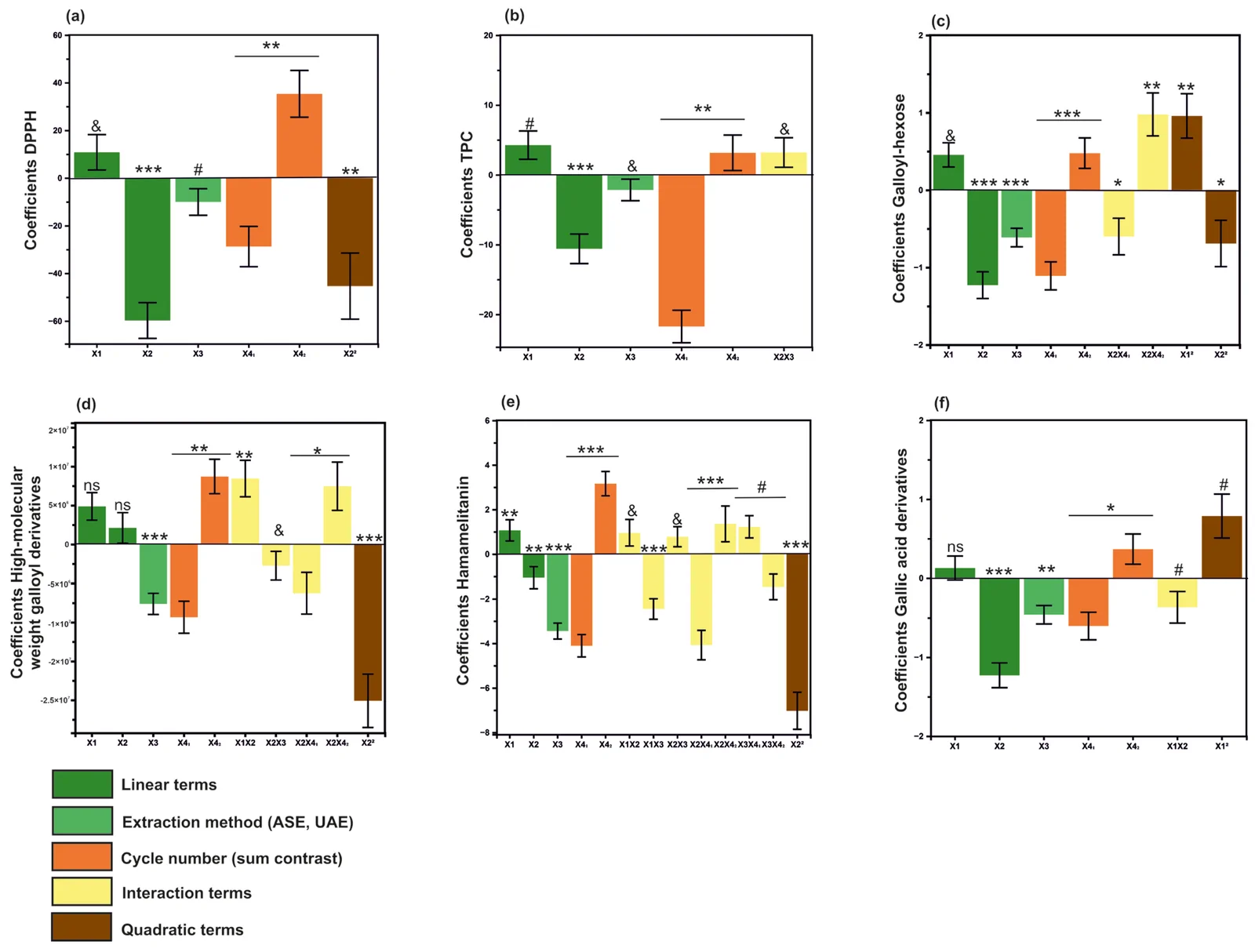
Regression coefficient plots of the fitted RSM models for bark extracts: (**a**) DPPH radical-scavenging activity, (**b**) total phenolic content (TPC), (**c**) galloyl-hexose, (**d**) high-molecular-weight galloyl derivatives, (**e**) hamamelitannin, and (**f**) gallic acid derivatives. X1—extraction temperature, X2—methanol concentration (%), X3—extraction technique (ASE or UAE), and X4—number of extraction cycles. Dark green bars represent linear terms, light green bars represent the effect of extraction technique, orange bars represent the number-of-cycles effect coded using sum contrasts, yellow bars represent interaction terms, and brown bars represent quadratic terms. Error bars indicate the uncertainty associated with the estimated regression coefficients. ns—*p* ≥ 0.15; & *p* < 0.15; # *p* < 0.10; * *p* < 0.05; ** *p* < 0.01; *** *p* < 0.001. The symbols indicate different *p*-value ranges for descriptive purposes only. Statistical significance was assessed at *p* < 0.05, and terms exceeding this threshold were not interpreted as statistically significant.

The regression coefficient and one-factor plots demonstrated that the extraction responses were strongly dependent on both the plant matrix and the chemical class considered. Overall, methanol concentration (X2) and the number of extraction cycles (X4) were among the most influential factors, whereas the effects of temperature (X1) and extraction technique (X3) were more response-specific (Figure 1 and Figure 2 and Supplementary Figures S4 and S5). The occurrence of several significant interaction and quadratic terms (*p* < 0.05), particularly those involving MeOH concentration and cycle number, confirmed that the extraction behavior was markedly nonlinear. The model fit statistics and significance tests are summarized in Table 1.

The developed models were statistically significant for all responses in both plant matrices. For leaf extraction, model *p*-values were < 0.0001 for all responses except hamamelitannin (*p* = 0.0025), while all bark models were highly significant (*p* < 0.0001) (Table 1). The coefficients of determination indicated generally good to very good agreement between the experimental and model-predicted data. For leaves, R^2^ values ranged from 0.812 for hamamelitannin to 0.983 for high-molecular-weight galloyl derivatives, whereas for bark they ranged from 0.857 for gallic acid derivatives to 0.973 for hamamelitannin. The adjusted R^2^ values remained relatively high (0.668–0.958 for leaves and 0.794–0.939 for bark), confirming that the selected model terms accounted for a substantial proportion of the observed variability without excessive overfitting.

The predicted R^2^ values further demonstrated satisfactory predictive performance for most responses (Table 1). For leaf extracts, predicted R^2^ ranged from 0.442 to 0.939. Particularly strong predictive ability was observed for TPC (0.939), DPPH (0.853), sum of flavonoids (0.803), gallic acid derivatives (0.808), and high-molecular-weight galloyl derivatives (0.837). The lower predicted R^2^ for hamamelitannin (0.442), together with a difference of more than 0.2 between adjusted and predicted R^2^, indicates comparatively weaker predictive robustness for this response and suggests that predictions outside the immediate experimental region should be interpreted with caution. For bark extracts, predicted R^2^ values ranged from 0.659 to 0.814 and were reasonably consistent with the corresponding adjusted R^2^ values, indicating acceptable model predictability across all responses. In addition, all Adequate Precision values were well above the recommended threshold of 4, ranging from 9.18 to 22.05 for leaves and from 10.99 to 20.44 for bark, confirming an adequate signal-to-noise ratio and the suitability of the models for navigating the experimental design space.

The lack-of-fit test was not significant (*p* > 0.05) for all leaf models, although values for hamamelitannin (*p* = 0.0703), ellagic acid (*p* = 0.0820), and gallic acid derivatives (*p* = 0.054) were relatively close to the significance threshold (Table 1). Similarly, no significant lack of fit was observed for most bark responses, with *p*-values ranging from 0.090 to 0.942. An exception was the model for high-molecular-weight galloyl derivatives in bark, for which the lack of fit was significant (*p* = 0.033). This result indicates that, despite the relatively high R^2^ (0.898) and adequate precision (10.99), the model may not fully capture the systematic variation in this response and therefore should be interpreted with greater caution than the remaining models.

For leaf extracts, increasing temperature generally favored extraction efficiency, with particularly pronounced positive effects on DPPH activity, flavonoids, and high-molecular-weight galloyl derivatives (Figure 1, Supplementary Figure S4). In contrast, the effect of MeOH concentration was strongly compound-dependent. Higher MeOH levels promoted the extraction of ellagic acid and flavonoids and, within part of the investigated range, high-molecular-weight galloyl derivatives, whereas gallic acid derivatives and hamamelitannin showed an opposite tendency. The quadratic terms observed for several responses were consistent with the curved one-factor profiles and indicated an optimum at intermediate solvent composition. Extraction technique also affected several phenolic groups, with ASE generally favoring the recovery of hamamelitannin, flavonoids, gallic acid derivatives, and high-molecular-weight galloyl derivatives compared with UAE, whereas its effect on DPPH activity was comparatively small. The effect of the number of extraction cycles was also response-dependent. Several responses reached or approached their highest predicted values after two extraction cycles, whereas TPC and some individual compounds showed further or sustained increases at three cycles.

A somewhat different pattern was observed for bark extracts, for which MeOH concentration exerted a particularly strong influence (Figure 2, Supplementary Figure S5). Increasing MeOH produced significant negative linear effects on several responses, including TPC and gallic acid derivatives, while DPPH activity, hamamelitannin, and high-molecular-weight galloyl derivatives displayed pronounced curvature, with the highest predicted values generally occurring at intermediate MeOH concentrations. Temperature had a comparatively smaller effect, although increasing temperature favored several responses, most notably hamamelitannin and, to a lesser extent, TPC and DPPH activity. The one-factor profiles also indicated that ASE was generally more favorable than UAE for bark extraction, particularly for hamamelitannin and several galloyl derivatives. As observed for leaves, the effect of cycle number was response-dependent. DPPH activity, hamamelitannin, gallic acid derivatives, and high-molecular-weight galloyl derivatives generally reached or approached their highest predicted values after two cycles, whereas TPC and galloyl-hexose tended to increase further with increasing cycle number.

Overall, the differences observed among individual responses and between leaves and bark indicated that no single combination of extraction parameters could simultaneously maximize all analytical responses, providing the rationale for subsequent multi-response optimization using the desirability function.

### 2.3. Multi-Response Optimization of Extraction Conditions

**Figure 3:**
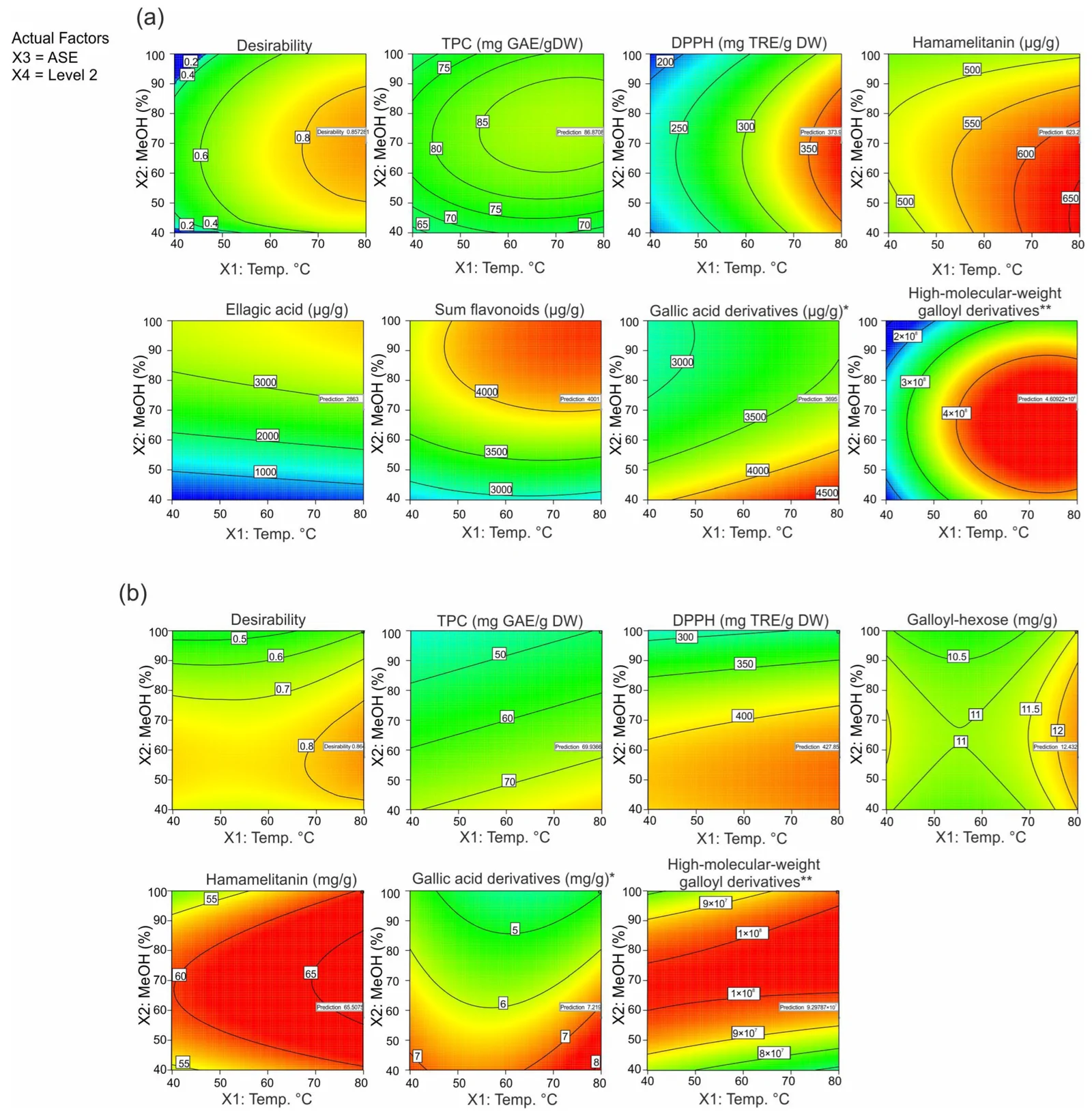
Response contour plots generated from the fitted DoE models for the multi-response optimization of accelerated solvent extraction (ASE). The effects of extraction temperature (X1) and methanol concentration (X2) on the predicted responses and overall desirability are shown. (**a**) Leaf extract and (**b**) bark extract of *Hamamelis virginiana*. The individual plots illustrate the effects of extraction conditions on the measured phytochemical and antioxidant-response variables. * Sum of gallic acid, galloylquinic acid isomers, and di-O-galloylquinic acid; ** Sum of the peak areas of selected high-molecular-weight galloylated compounds, including tri-, tetra-, penta-, hexa-, hepta-, and octa-O-galloyl-hexoses.

**Figure 4:**
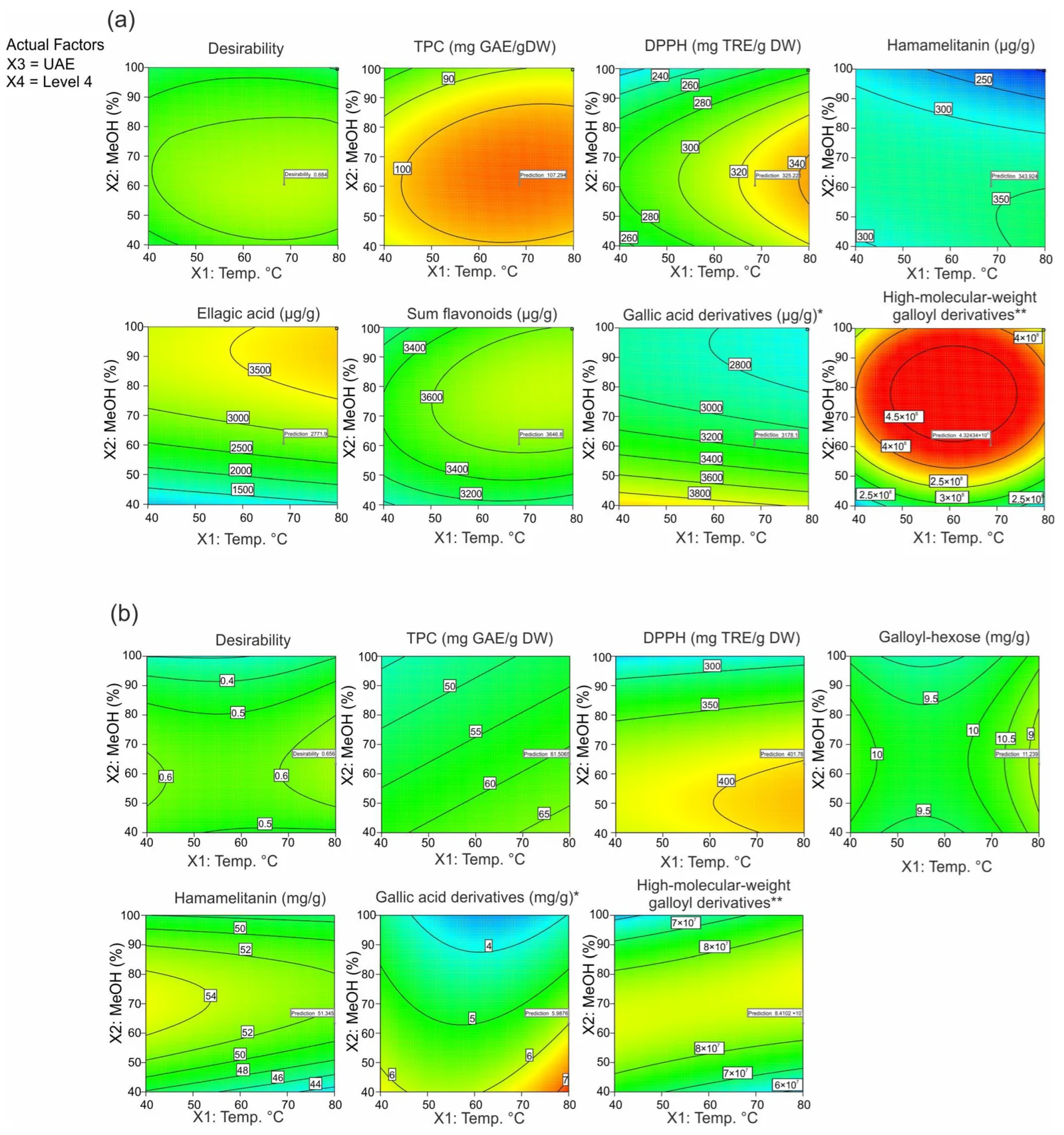
Response contour plots generated from the fitted DoE models for the multi-response optimization of ultrasound-assisted extraction (UAE). The effects of extraction temperature (X1) and methanol concentration (X2) on the predicted responses and overall desirability are shown. (**a**) Leaf extract and (**b**) bark extract of *Hamamelis virginiana*. The individual plots illustrate the effects of extraction conditions on the measured phytochemical and antioxidant-response variables. * Sum of gallic acid, galloylquinic acid isomers, and di-O-galloylquinic acid; ** Sum of the peak areas of selected high-molecular-weight galloylated compounds, including tri-, tetra-, penta-, hexa-, hepta-, and octa-O-galloyl-hexoses.

Because the individual analytical responses showed different dependencies on the investigated extraction parameters, simultaneous multi-response optimization was performed using the desirability function. The optimization criteria applied to leaf and bark extracts are presented in Supplementary Tables S6 and S7, respectively. Optimization was conducted separately for ASE and UAE and for both plant matrices. Response contour plots were generated to visualize the effects of extraction temperature and methanol concentration on the individual responses and overall desirability under the selected number of extraction cycles (Figure 3 and Figure 4).

For ASE, the overall desirability showed a clear dependence on solvent composition and plant matrix (Figure 3). For leaf extracts, the highest overall desirability was observed at approximately 70% MeOH, whereas for bark extracts the maximum was shifted toward lower MeOH concentrations, approximately 55–60%. In both matrices, higher extraction temperatures were associated with higher overall desirability. The multi-response optimization predicted optimal ASE conditions of 80 °C, 70.3% MeOH, and two extraction cycles for leaves and 80 °C, 57.5% MeOH, and two extraction cycles for bark.

For UAE, the overall desirability also showed a clear dependence on extraction temperature and solvent composition, although the response patterns differed between the two plant matrices (Figure 4). For leaf extracts, the region of highest overall desirability was observed at intermediate methanol concentrations and relatively high extraction temperatures, with the predicted optimum located at 72 °C and 63.5% MeOH. For bark extracts, overall desirability increased toward higher extraction temperatures, while the most favorable solvent composition was centered at approximately 60–65% MeOH. The corresponding optimal numbers of extraction cycles were three for leaves and two for bark.

Overall, the optimal extraction conditions were both matrix- and technique-dependent. The optimal methanol concentration ranged from 57.5 to 70.3%, with the lowest value predicted for ASE of bark and the highest for ASE of leaves.

### 2.4. Experimental Validation of the Optimized Conditions

The predictive performance of the fitted models was evaluated experimentally under the optimized extraction conditions. Confirmation experiments were performed in triplicate, and the experimentally obtained values were compared with the model-predicted responses and the corresponding 95% prediction intervals (Table 2). The experimental validation showed varying degrees of agreement between the predicted and observed responses, depending on the analytical parameter, extraction technique, and plant matrix. All experimentally observed values, except TPC for leaf UAE, fell within the corresponding 95% prediction intervals. For leaf UAE, the observed TPC (96.3 mg GAE/g DW) was slightly below the lower prediction limit (98.2 mg GAE/g DW), indicating a minor discrepancy between the model prediction and experimental validation. Furthermore, the predominance of positive relative deviations suggested a tendency toward overprediction. For leaf extracts, the relative deviations (RD) between the predicted and experimentally observed values ranged from 5.7 to 20.1% for ASE and from −8.7 to 20.7% for UAE. For bark extracts, the corresponding RD values ranged from −5.5 to 9.7% for ASE and from 1.9 to 15.4% for UAE. Overall, these findings indicate that, although the models provided reasonable estimates of extraction performance under the optimized conditions, their predictive accuracy varied among individual responses. Therefore, the validation results should be interpreted with consideration of both the magnitude and direction of prediction errors and the uncertainty reflected by the prediction intervals.

## 3. Discussion

*Hamamelis virginiana* is a medicinal plant with considerable potential for cosmetic applications, largely attributed to its rich content of biologically active phenolic compounds [19]. Previous studies have demonstrated that both its leaves and bark are abundant sources of hydrolysable tannins, proanthocyanidins, flavonoids, and phenolic acids [3,7,19]. The abundance and diversity of phenolic constituents were also observed in the present study. The profile of the major metabolites was generally consistent with that observed in our previous work and the literature data [3,7,9,19], although some differences in the occurrence of individual compounds were noted. Most notably, myricetin 3-*O*-rhamnoside was identified as the predominant flavonoid in the leaf extracts, whereas it was not detected previously. The plant materials analyzed in the two studies originated from different batches, and this discrepancy may therefore reflect natural batch-to-batch variability in the phytochemical composition. Such variability is common in plant-derived materials and may arise from differences in environmental and seasonal conditions or harvesting time [20,21]. Importantly, the presence of myricetin derivatives in *H. virginiana* leaves has also been reported [22], supporting its occurrence as a constituent of this species.

The potential utilization of plant material depends not only on its phytochemical composition but also on the efficient recovery of bioactive constituents from the plant matrix. The extraction process can be strongly affected by the physicochemical properties of the target compounds, the extraction technique, and the conditions applied [23,24]. In the present study, accelerated solvent extraction (ASE) and ultrasound-assisted extraction (UAE) were investigated as alternative approaches for the recovery of phenolic compounds from *H. virginiana* bark and leaves. A Design-of-Experiments (DoE) approach was employed to systematically assess the effects of the investigated process parameters and their interactions. This strategy offers advantages over the conventional one-factor-at-a-time (OFAT) approach, which does not account for interactions between the investigated factors. The usefulness of DoE for optimizing the extraction of phenolic compounds from plant materials has been widely demonstrated in the literature [25,26]; however, to the best of our knowledge, this approach has not yet been applied to *H. virginiana*. Various extraction solvents, including water, aqueous ethanol, methanol, and acetone–water mixtures, as well as different extraction techniques, such as maceration, UAE, HRE, and ASE, have been employed for the recovery of phenolic constituents from *H. virginiana* [3,7,8]. However, these studies have generally relied on predefined extraction conditions or direct comparisons of selected extraction procedures rather than systematic multivariable optimization.

Our study revealed that extraction efficiency was dependent not only on the extraction technique and process variables but also on the plant matrix. The pronounced effect of solvent composition observed in the present study can be related to differences in the polarity and structural properties of the extracted phenolic constituents. Aqueous methanol mixtures provide a broad polarity range, and changes in the methanol-to-water ratio may therefore selectively favor phenolic compounds with different structural features and polarities [27,28]. This was reflected in the markedly different responses of individual phenolic groups to changes in methanol concentration. In leaf extracts, higher methanol concentrations favored the recovery of ellagic acid and flavonoids, whereas gallic acid derivatives and hamamelitannin showed an opposite tendency. Similarly, the nonlinear responses observed for several constituents in bark indicate that intermediate methanol concentrations provided a more favorable balance between solvent polarity and the extraction of chemically diverse phenolic compounds.

Methanol–water mixtures were selected for the present study to investigate the extraction behavior of chemically diverse phenolic compounds under controlled analytical conditions. However, methanol is not suitable for direct use in food or cosmetic formulations. Although ethanol–water mixtures represent a more appropriate alternative for practical applications, differences in solvent properties may affect extraction efficiency and selectivity. Therefore, the optimized conditions established in this study cannot be directly transferred to ethanol-based extraction and require separate experimental verification.

Extraction temperature and the number of extraction cycles also affected extraction performance, although their effects were response-dependent. Increasing temperature can enhance extraction by improving solvent penetration into the plant matrix and accelerating mass transfer, while reducing solvent viscosity and surface tension [12,14]. In the present study, higher temperatures generally favored several responses, although the extent of this effect differed between phenolic groups and plant matrices. Moreover, three of the four optimized extraction temperatures were located at the upper boundary of the experimental design (80 °C), indicating that further increases in some analytical responses beyond the investigated temperature range cannot be excluded. Therefore, the identified conditions should be regarded as optimal within the studied experimental domain rather than as absolute optima. The number of extraction cycles was also an important factor, with several responses reaching or approaching their highest predicted values after two cycles. The limited additional increase observed for several responses after a third cycle suggests that a substantial proportion of the readily extractable phenolic fraction had already been recovered during the preceding extraction steps. The effect of extraction technique was likewise dependent on the analytical response and plant matrix. ASE generally favored the recovery of several phenolic groups compared with UAE, particularly hamamelitannin and selected galloyl derivatives, although this trend was not uniform across all responses. The differences between the two techniques may be related to their distinct extraction mechanisms. ASE combines elevated temperature and pressure, facilitating solvent penetration and mass transfer, whereas UAE relies primarily on acoustic cavitation to disrupt the plant matrix and enhance solvent–sample interactions [10,12]. Importantly, the present results do not indicate the universal superiority of either technique but rather demonstrate that their relative performance depends on the targeted compounds and plant matrix. In addition to extraction efficiency, practical aspects such as extraction time and solvent consumption should be considered when comparing ASE and UAE. Under the optimized conditions, ASE required 10 min of static extraction (two cycles of 5 min each), compared with 30–45 min of sonication for UAE. Although solvent consumption was comparable between the two techniques, ASE offered shorter nominal extraction times. However, additional operational steps, including heating and pressurization, should be considered when evaluating the overall processing time.

A particularly relevant finding of the present study was the clear matrix dependence of the extraction process. The optimized conditions differed between leaves and bark, most notably in terms of solvent composition. For ASE, the optimal methanol concentration was 70.3% for leaves compared with 57.5% for bark, while the corresponding values for UAE were 63.5% and 63.3%, respectively. These differences are consistent with the distinct phytochemical profiles of the two plant materials. Leaves contained a broader range of flavonol glycosides and a greater number of highly galloylated, high-molecular-weight compounds, whereas bark was characterized predominantly by lower-molecular-weight galloyl derivatives. The different chemical composition of the two matrices likely contributed to their distinct responses to changes in extraction conditions.

Several limitations of the present study should be acknowledged. First, three batches of plant material were pooled before analysis to obtain a composite sample. Although this approach enabled the evaluation of the combined material, it prevented the assessment of batch-to-batch variability. Second, high-molecular-weight galloyl derivatives were evaluated using chromatographic peak areas because authentic reference standards were unavailable. Consequently, the obtained values represent chromatographic responses rather than absolute quantities of these compounds. An important limitation is that the extraction procedure factor (X3) encompasses differences not only in extraction mechanisms but also in operational parameters, particularly extraction time and pressure. Consequently, the higher extraction efficiency observed for ASE should be attributed to the overall extraction procedure under the investigated conditions rather than to the extraction technique alone. Additional limitations are associated with the developed response surface models. The model describing hamamelitannin recovery from leaf extracts exhibited relatively limited predictive performance (predicted R^2^ = 0.442), while the model for high-molecular-weight galloyl derivatives in bark extracts showed a statistically significant lack of fit (*p* = 0.033). These findings indicate increased uncertainty in predicting the corresponding responses. Although experimental validation under the optimized conditions demonstrated reasonable agreement between predicted and observed values, some 95% prediction intervals were relatively wide, particularly for ellagic acid in leaf extracts. The observed TPC for leaf UAE was slightly below the lower 95% prediction limit, suggesting some uncertainty in the model prediction for this response. Moreover, the observed deviations between experimental and predicted values indicate that the models do not provide uniformly high predictive accuracy. Both responses were included as components of the multi-response optimization rather than used independently to determine the optimal extraction conditions. Therefore, the optimized conditions should be regarded as a practical compromise among the investigated analytical responses, and the corresponding model predictions should be interpreted with caution within the studied experimental domain.

Our findings highlight the complexity of optimizing the extraction of chemically diverse plant metabolites and show that a single set of conditions cannot necessarily maximize the recovery of all phenolic constituents. Therefore, the effects of the individual extraction parameters and their interactions should be considered in relation to both the overall properties of the obtained extracts and the extraction behavior of specific compounds.

## 4. Materials and Methods

### 4.1. Plant Material

Both the bark and leaves of *Hamamelis virginiana* L. were purchased from a Polish supplier, Plantago (Dzierzążenko, Poland), in three separate batches. The individual batches were combined to ensure sample homogeneity and then ground into a fine powder using a laboratory mill (IKA M20, IKA-Werke GmbH & Co. KG, Staufen, Germany).

### 4.2. Experimental Design and Optimization of Extraction Conditions Using RSM

The extraction conditions were optimized using response surface methodology (RSM) to evaluate the effects of four independent variables on the extraction efficiency of phenolic compounds and the antioxidant activity of leaf and bark samples. The investigated factors were extraction temperature (X1, 40–80 °C), methanol concentration in the extraction solvent (X2, 40–100%, *v*/*v*), extraction method (X3; accelerated solvent extraction, ASE, or ultrasound-assisted extraction, UAE), and number of extraction cycles (X4; 1–3). The ranges of the numerical variables were selected on the basis of preliminary experiments and practical limitations of the extraction procedures. The number of extraction cycles was treated as a discrete numerical factor, whereas the extraction technique was included as a categorical factor.

Because the experimental system included both numerical and categorical variables, a mixed-factor response surface design was applied. A custom I-optimal design was generated to minimize the average prediction variance across the investigated experimental domain while providing efficient estimation of the main, interaction, and curvature effects. The experimental runs were performed in a randomized order to minimize potential systematic bias associated with uncontrolled experimental factors. The experimental design included a replicated point at 55 °C, 65% methanol (*v*/*v*), using ASE with three extraction cycles. This experimental condition was independently repeated three times to provide an estimate of pure experimental error. The pure-error variance estimated from these replicates was used in the analysis of variance (ANOVA) to evaluate the lack of fit of the fitted regression models. Polynomial models were developed independently for each response and separately for leaf and bark samples. For the numerical factors, linear, two-factor interaction, and, where statistically justified, quadratic terms were considered, whereas the effect of the categorical extraction method and its interactions with the numerical factors were also evaluated.

For leaf samples, the responses used for model development and optimization were total phenolic content (TPC, mg GaE/g DW), DPPH radical-scavenging activity (mg TRE/g DW), hamamelitannin content (µg/g), ellagic acid content (µg/g), sum of flavonoids (µg/g), gallic acid derivatives (µg/g), and high-molecular-weight galloyl derivatives. For bark samples, the evaluated responses included TPC (mg GaE/g DW), DPPH radical-scavenging activity (mg TRE/g DW), galloyl-hexose content (mg/g), hamamelitannin content (mg/g), gallic acid derivatives (mg/g), and high-molecular-weight galloyl derivatives.

Potentially anomalous observations were evaluated using regression diagnostic tools implemented in Design-Expert software (version 13, Stat-Ease Inc., Minneapolis, MN, USA). The diagnostic assessment included externally studentized residuals, used to identify observations showing substantial deviations from model predictions, and Cook’s distance, used to evaluate the influence of individual observations on the fitted regression models. Observations identified as potentially influential were individually examined before exclusion from model fitting. A total of seven response-specific observations were excluded: five for leaf extracts (one for chlorogenic acid, two for methyl gallate, and two for the sum of galloyl-hexoses) and two for bark extracts (one for catechin and one for DPPH radical-scavenging activity). Exclusions were applied only to the affected analytical responses, while the remaining data from the corresponding experimental runs were retained.

The significance of each fitted model and individual model terms was assessed by analysis of variance (ANOVA). Model adequacy and predictive performance were evaluated using the coefficient of determination (R^2^), adjusted R^2^, predicted R^2^, adequate precision, and the lack-of-fit test.

The optimization procedure was performed separately for leaf and bark samples because of differences in their chemical composition and extraction behavior. Multiple-response optimization was carried out using the Derringer–Suich desirability function approach. Each experimental response was transformed into an individual desirability function (d_i_), ranging from 0, representing a completely undesirable result, to 1, representing the most desirable result. The overall desirability (D) was calculated as the geometric mean of the individual desirability values. The optimization criteria, including the goals, lower and upper limits, weights, and relative importance assigned to each response, were specified separately for leaf and bark extracts. The optimization was performed independently for ASE and UAE to identify extraction conditions providing the most favorable combination of the investigated analytical responses. Detailed settings used for the desirability-based optimization are summarized in Supplementary Tables S6 and S7.

The predictive capability of the developed models was verified by confirmation experiments performed under the conditions predicted to be optimal for leaves and bark. Each confirmation experiment was carried out in triplicate (*n* = 3), and the experimentally obtained values were compared with the model-predicted responses. Model accuracy was assessed using relative deviation (RD, %) and the agreement of the experimental values with the 95% prediction intervals generated by the models.

Relative deviation was calculated as follows:

$$\mathrm{RD}\left(\%\right)=\frac{Y_{\text{observed}}\;-\;Y_{\text{predicted}}}{Y_{\text{predicted}}}\times 100$$

Good predictive performance was considered to be indicated by low deviations between predicted and experimental values and by experimental results falling within the corresponding 95% prediction intervals.

Experimental design generation, regression modelling, ANOVA, response surface analysis, desirability-based optimization, and calculation of prediction intervals were performed using Design-Expert software, version 13 (Stat-Ease Inc., Minneapolis, MN, USA).

### 4.3. Extraction Conditions

#### 4.3.1. Accelerated Solvent Extraction (ASE)

Approximately 250 mg of the plant material was weighed using an analytical balance (XA105DU, Mettler Toledo, Greifensee, Switzerland) and quantitatively transferred to 5 mL stainless-steel extraction cells (Dionex™). Accelerated solvent extraction (ASE) was performed using a Dionex™ ASE™ 350 system (Thermo Fisher Scientific Inc., Sunnyvale, CA, USA), with a static extraction time of 5 min, within a Design-of-Experiments (DoE) framework. The samples were extracted under variable conditions, including different numbers of extraction cycles (1–3), extraction temperatures (40–80 °C), and different proportions of the methanol–water mixture, as presented in Table 3. The extracts obtained from the successive extraction cycles were combined and then adjusted with the appropriate extraction solvent to a final volume of 10 mL for one extraction cycle, 20 mL for two cycles, and 30 mL for three cycles. The resulting extracts were subsequently filtered through 0.22 µm nylon filters prior to further analysis.

#### 4.3.2. Ultrasound-Assisted Extraction (UAE)

Approximately 250 mg of the plant material was weighed using an analytical balance (XA105DU, Mettler Toledo, Greifensee, Switzerland) and quantitatively transferred to 50 mL Falcon tubes, to which 10 mL of the appropriate extraction mixture was added. The extraction was performed using an ultrasonic bath (RK 102H, Sonorex, Bandelin, Berlin, Germany) with a water circulation system in capped Falcon tubes, following the procedure described by Dresler et al. [29] within a Design-of-Experiments (DoE) framework. The bath temperature was monitored using a thermometer during extraction.

The samples were extracted under variable conditions, including different numbers of extraction cycles (1–3, each cycle lasting 15 min), extraction temperatures (40–80 °C), and different proportions of the methanol–water mixture, as presented in Table 3.

After each extraction cycle, the samples were centrifuged in a laboratory centrifuge (MPW-352R, MPW Med. Instruments, Warsaw, Poland) for 10 min at 5200 rpm. The supernatant was then collected and adjusted to a volume of 10 mL with the appropriate extraction solvent for a single extraction cycle. For two or three extraction cycles, the remaining plant material was re-extracted with a fresh portion of the appropriate extraction solvent after each cycle. The supernatants obtained from the successive extraction cycles were combined and adjusted to a final volume of 20 mL for two cycles and 30 mL for three cycles using the respective extraction solvent. The resulting extracts were then filtered through 0.22 µm nylon filters prior to further analysis.

### 4.4. Phytochemical Analysis

An Infinity Series II ultra-high-performance liquid chromatograph (UHPLC) equipped with a DAD detector and coupled to an Agilent 6224 ESI/TOF mass spectrometer (Agilent Technologies, Santa Clara, CA, USA) was used for chromatographic analysis. Separation was performed on a Kinetex C18 column (150 × 2.1 mm, 1.7 µm particle size; Phenomenex, Torrance, CA, USA) maintained at 30 °C. The mobile phase consisted of water containing 0.05% formic acid (solvent A) and acetonitrile containing 0.05% formic acid (solvent B), delivered at a flow rate of 0.2 mL/min. The following gradient elution program was applied: 0–8 min, 98–93% A; 8–15 min, 93–88% A; 15–29 min, 88–85% A; 29–40 min, 85–80% A; and 40–80 min, 80–55% A. The ESI/TOF-MS conditions were as follows: drying gas temperature, 325 °C; drying gas flow rate, 8 L/min; nebulizer pressure, 30 psi; capillary voltage, 3500 V; and skimmer voltage, 65 V. The fragmentor voltage was set at 180 and 280 V, and data were acquired in negative-ion mode. Compounds were tentatively identified based on their UV–Vis spectra and accurate mass measurements, supported by comparison with authentic standards when available or with the previously reported literature data.

Quantification was performed using DAD detection and external calibration. Calibration curves were constructed using authentic reference standards when available. For compounds for which reference standards were unavailable, structurally related compounds exhibiting similar spectral properties were used for quantification, with appropriate molecular-mass correction. Detailed information is provided in the Supplementary Materials (Table S1). The calibration curve parameters for the analyzed compounds are provided in Supplementary Table S8.

### 4.5. Determination of Total Phenolic Content and DPPH Radical-Scavenging Activity

The TPC assay was performed according to the procedure described previously [30]. For the determination of total phenolic content (TPC), 25 μL of the diluted extracts were mixed with 25 μL of ninefold-diluted Folin–Ciocalteu (FC) reagent and 200 μL of distilled water in each well. After 5 min of incubation, 25 μL of saturated sodium carbonate (Na_2_CO_3_) solution was added. The microplate was then incubated in the dark at room temperature for 60 min. Absorbance was measured in a microplate reader (BioTek Epoch, Agilent Technologies, Santa Clara, CA, USA) at a wavelength of λ = 750 nm. The gallic acid (Merck KGaA, Darmstadt, Germany) calibration curve was prepared in a range of concentrations (0–0.2 mg/mL). The results are presented as the equivalent of mg of gallic acid per gram of dry weight (DW). To evaluate the free radical scavenging capacity of the extracts, a test was conducted using the DPPH (1,1-diphenyl-2-picrylhydrazyl) free radical [31]. For this purpose, samples were prepared at appropriate dilutions. Next, 50 µL of the diluted extracts were dispensed into 96-well plates, after which 200 µL of 0.2 mM DPPH in methanol (Merck KGaA, Darmstadt, Germany) was added to each well and mixed on an orbital shaker. The control sample consisted of DPPH solution and methanol. The Trolox (Merck KGaA, Darmstadt, Germany) standard curve was prepared in a range of concentrations (0–300 μM). After incubation in the dark for 30 min at room temperature, absorbance was measured in a microplate reader (BioTek Epoch, Agilent Technologies, Santa Clara, CA, USA) at a wavelength of λ = 517 nm. The results are presented as the equivalent of mg of Trolox per gram of dry weight (DW).

## 5. Conclusions

This study demonstrated the usefulness of a Design-of-Experiments approach for optimizing the extraction of phenolic compounds from *Hamamelis virginiana* leaves and bark using accelerated solvent extraction (ASE) and ultrasound-assisted extraction (UAE). Extraction efficiency was influenced by solvent composition, temperature, extraction technique, and the number of extraction cycles, although their effects differed among individual analytical responses and between the two plant matrices. In particular, methanol concentration and the number of extraction cycles were identified as important factors under the investigated experimental conditions.

Multi-response optimization showed that no single set of extraction conditions was optimal for all investigated responses and that the optimal conditions differed between leaves and bark as well as between ASE and UAE. Within the investigated experimental ranges, the predicted optimal conditions were 80 °C, 70.3% MeOH, and two cycles for ASE of leaves and 80 °C, 57.5% MeOH, and two cycles for ASE of bark, whereas UAE was optimized at 72 °C, 63.5% MeOH, and three cycles for leaves and 80 °C, 63.3% MeOH, and two cycles for bark.

Overall, the results indicate that optimization of phenolic compound extraction should consider both the plant matrix and the targeted chemical constituents. The developed DoE-based approach provides a rational basis for selecting ASE and UAE conditions. However, the optimized conditions are specific to the investigated composite plant material and methanol–water extraction system. Further studies involving independent plant batches and ethanol–water mixtures are required to evaluate their broader applicability and potential implementation in pharmaceutical and cosmetic extraction processes.

## Figures and Tables

**Table 1:** Model fit statistics and significance tests.

Response	Model *p*-Value	Lack-of-Fit *p*-Value	R^2^	Adj. R^2^	Pred. R^2^	Adeq. Prec.
Leaves extraction						
TPC	<0.0001	0.7637	0.980	0.958	0.939	18.38
DPPH	<0.0001	0.4507	0.964	0.932	0.853	22.05
Hamamelitannin	0.0025	0.0703	0.812	0.668	0.442	9.18
Ellagic acid	<0.0001	0.0820	0.879	0.826	0.722	14.48
Sum flavonoids	<0.0001	0.761	0.965	0.932	0.803	20.28
Gallic acid derivatives	<0.0001	0.054	0.912	0.873	0.808	20.76
High-molecular-weight galloyl derivatives	<0.0001	0.340	0.983	0.955	0.837	19.85
Bark extraction						
TPC	<0.0001	0.502	0.903	0.869	0.814	18.19
DPPH	<0.0001	0.942	0.861	0.809	0.721	12.928
Galloyl-hexose	<0.0001	0.673	0.930	0.885	0.791	17.937
Hamamelitannin	<0.0001	0.608	0.973	0.939	0.799	20.44
Gallic acid derivatives	<0.0001	0.090	0.857	0.794	0.659	15.37
High-molecular-weight galloyl derivatives	<0.0001	0.033	0.898	0.820	0.660	10.99

**Table 2:** Predicted value and experimental mean values (±SD, *n* = 3) at the optimal extraction conditions for ASE and UAE methods.

	Leaves extraction									
	ASE method					UAE method				
	Temp.: 80 °C; 70.3% MeOH; ASE; 2 cycles					Temp.: 72 °C; 63.5% MeOH; UAE; 3 cycles				
Analysis	Predicted Mean	95% PI low	Observed mean value ± SD	95% PI high	RD%	Predicted Mean	95% PI low	Observed mean value ± SD	95% PI high	RD%
TPC (mg GAE/g DW)	86.9	75.9	80.0 ± 2.45	97.9	8.6	107.4	98.2	96.3 ± 2.56	116.6	11.5
DPPH (mg TRE/g DW)	373.9	343.4	344.5 ± 18.07	404.5	8.5	330.8	308.8	335.5 ± 12.57	352.8	−1.4
Hamamelitannin (µg/g)	623.2	483.6	560.9 ± 5.24	762.8	11.1	341.1	220.4	282.7 ± 12.08	461.9	20.7
Ellagic acid (µg/g)	2863.1	1917.0	2383.6 ± 69.48	3809.2	20.1	2957.0	2122.5	3238.1 ± 139.3	3791.5	−8.7
Sum flavonoids (µg/g)	4001.1	3730.5	3753.6 ± 28.06	4271.8	6.6	3689.8	3457.7	3512.0 ± 107.6	3922.0	5.1
Gallic acid derivatives (µg/g) *	3695.3	3291.3	3495.3 ± 57.70	4099.3	5.7	3092.3	2735.0	2856.3 ± 152.7	3449.7	8.3
High-molecular-weight galloyl derivatives **	4.61 × 10^8^	4.13 × 10^8^	4.25 × 10^8^ ± 6.72× 10^6^	5.09 × 10^8^	8.4	4.32 × 10^8^	3.99 × 10^8^	4.04 × 10^8^ ± 1.33 × 10^7^	4.66 × 10^8^	7.0
	Bark extraction									
	Temp.: 80 °C; 57.5% MeOH; ASE; 2 cycles					Temp.: 80 °C; 63.3% MeOH; UAE; 2 cycles				
TPC (mg GAE/g DW)	70.0	56.6	70.9 ± 0.41	83.3	−1.4	61.5	48.3	57.0 ± 2.25	74.7	8.0
DPPH (mg TRE/g DW)	427.9	378.7	431.6 ± 0.99	477.1	−0.8	401.8	352.2	377.9 ± 11.77	451.3	6.3
Galloyl-hexose (mg/g)	12.4	11.3	13.1 ± 0.11	13.6	−5.5	11.2	10.1	10.7 ± 0.47	12.4	5.3
Hamamelitannin (mg/g)	65.5	61.6	65.6 ± 0.73	69.4	−0.1	51.3	47.7	50.4 ± 1.01	55.0	1.9
Gallic acid derivatives (mg/g) *	7.2	6.2	6.6 ± 0.01	8.3	9.7	6.0	5.0	5.2 ± 0.16	7.0	15.4
High-molecular-weight galloyl derivatives **	9.29 × 10^7^	8.03 × 10^7^	9.04 × 10^7^ ± 1.48× 10^6^	1.06 × 10^8^	2.8	8.41 × 10^7^	7.18 × 10^7^	7.41 × 10^7^ ± 1.82× 10^6^	9.64 × 10^7^	13.6

* sum of gallic acid, galloylquinic acid isomers, and di-O-galloylquinic acid; ** Sum of the peak areas of selected high-molecular-weight galloylated compounds, including tri-, tetra-, penta-, hexa-, hepta-, and octa-O-galloyl-hexoses.

**Table 3:** Experimental factors used in the Design of Experiments (DoE) for ASE and UAE.

No.	Temperature (°C); X1	MeOH/Water (*v*/*v*); X2	Technique; X3	Number of Cycles; X4
1	40	100/0	ASE	3
2	40	40/60	ASE	1
3	40	40/60	UAE	3
4	40	45/55	ASE	3
5	40	75/25	UAE	1
6	40	80/20	ASE	2
7	45	45/55	ASE	2
8	45	70/30	UAE	3
9	45	90/10	UAE	2
10	55	100/0	ASE	1
11	55	100/0	UAE	3
12	55	65/35	ASE	3
13	60	50/50	ASE	1
14	60	50/50	UAE	3
15	65	40/60	UAE	1
16	75	50/50	UAE	2
17	75	95/5	ASE	2
18	80	100/0	ASE	3
19	80	100/0	UAE	1
20	80	40/60	ASE	3
21	80	60/40	UAE	3
22	80	65/35	ASE	1

## Data Availability

Raw, unprocessed data are available from the corresponding authors upon a reasonable request.

## Supplementary Materials

The following supporting information can be downloaded at https://www.mdpi.com/article/10.3390/molecules31213813/s1. Table S1: Summary of main compounds quantified in witch hazel bark and leaves, including the wavelengths used for quantification and the compounds used to construct the calibration curves; Table S2: Complete experimental design matrix and raw analytical responses obtained for *Hamamelis virginiana* leaf extracts; Table S3: Complete experimental design matrix and raw analytical responses obtained for *Hamamelis virginiana* bark extracts; Table S4: Analysis of variance (ANOVA) for the fitted response surface models of (a) total phenolic content (TPC), (b) DPPH, (c) hamamelitannin, (d) ellagic acid, (e) sum of flavonoids, (f) gallic acid derivatives, and (g) high-molecular-weight galloyl derivatives in *Hamamelis virginiana* leaf extracts; Table S5: Analysis of variance (ANOVA) for the fitted response surface models of (a) total phenolic content (TPC), (b) DPPH, (c) galloyl-hexose, (d) hamamelitannin, (e) gallic acid derivatives, and (f) high-molecular-weight galloyl derivatives in *Hamamelis virginiana* bark extracts; Table S6: Optimization criteria applied in the desirability function approach for the multi-response optimization of phenolic compound extraction from *Hamamelis virginiana* leaves; Table S7: Optimization criteria applied in the desirability function approach for the multi-response optimization of phenolic compound extraction from *Hamamelis virginiana* bark; Table S8: Calibration curve parameters for the analyzed compounds. Figure S1: Representative chromatograms of *Hamamelis virginiana* leaf (a) and bark (b) extracts; Figure S2: Relative contribution of individual flavonoids to the total quantified flavonoid content of H. virginiana leaf extracts. Values represent the mean ± SD calculated across the ten extraction conditions investigated; Figure S3: Extracted ion chromatograms (EICs) in the m/z ranges corresponding to galloylhexoses and high-molecular-weight tannins in H. virginiana leaf (green line) and bark (red line) extracts; Figure S4: One-factor plots illustrating the effect of individual extraction factors on the predicted responses and overall desirability for leaf extracts; Figure S5: One-factor plots illustrating the effect of individual extraction factors on the predicted responses and overall desirability for bark extracts.
