# Supplementary material for: Efficient Extraction of Polyphenols from Hamamelis virginiana Bark and Leaves: A Design-of-Experiments Approach to Accelerated Solvent- and Ultrasound-Assisted Extraction
Source: Molecules. 2026 Oct 22;31(21):3813. doi: 10.3390/molecules31213813 (PMC13765150; doi:10.3390/molecules31213813)
Supplement: Supplementary file 1 [file molecules-31-03813-s001.zip › molecules-4613531-supplementary.pdf]

Supplementary material

Efficient Extraction of Polyphenols from *Hamamelis virginiana* Bark and Leaves: A Design of Experiments Approach to Accelerated Solvent and Ultrasound-Assisted Extraction

Wiktoria Pacuła, Weronika Wójciak, Sławomir Dresler, Ireneusz Sowa, Agnieszka Skalska-Kamińska, Magdalena Wójciak

Table S1. Summary of main compounds quantified in witch hazel bark and leaves, including the wavelengths used for quantification and the compounds used to construct the calibration curves.

RT (min.)	Mass data (m/z-H)	Formula	Component	B	L	Wavelength (nm)	Calibration*
3.88	331.06762	$C_{13}H_{16}O_{10}$	Galloyl-hexose	+	-	270	Gallic acid
4.81	169.01488	$C_7H_6O_5$	Gallic acid	+	+	270	standard
6.04	343.06652	$C_{14}H_{16}O_{10}$	Galloylquinic acid	+	+	270	Gallic acid
7.10	331.06782	$C_{13}H_{16}O_{10}$	Galloyl-hexose	+	-	270	Gallic acid
14.87	495.07708	$C_{21}H_{20}O_{14}$	di-O-galloylquinic acid	+	-	270	Hamamelitannin
15.77	635.08906	$C_{27}H_{24}O_{18}$	Tri-O-galloyl-hexose	+	-	270	nq
16.37	483.07948	$C_{20}H_{20}O_{14}$	Hamamelitanin	+	+	270	standard
21.87	635.08889	$C_{27}H_{24}O_{18}$	Tri-O-galloyl-hexose	+	-	270	nq
26.13	787.09821	$C_{34}H_{28}O_{22}$	Tetra-O-galloylhexose	-	+	270	nq
30.20	787.09806	$C_{34}H_{28}O_{22}$	Tetra-O-galloylhexose	+	+	270	nq
30.66	300.99868	$C_{14}H_6O_8$	Ellagic acid	+	+	254	standard
31.45	463.08963	$C_{21}H_{20}O_{12}$	Myricetin 3-O-rhamnoside	-	+	350	standard
31.89	787.09883	$C_{34}H_{28}O_{22}$	Tetra-O-galloyl-hexose	+	+	270	nq
32.78	463.08856	$C_{21}H_{20}O_{12}$	Quercetin 3-O-galactoside	-	+	350	standard
33.58	477.06752	$C_{21}H_{18}O_{13}$	Quercetin-3-O-glucuronide	-	+	350	standard
33.93	463.08896	$C_{21}H_{20}O_{12}$	Quercetin 3-O-glucoside	-	+	350	standard
37.51	447.09362	$C_{21}H_{20}O_{11}$	Kaempferol 3-O-galactoside	-	+	350	standard
38.36	593.15178	$C_{27}H_{30}O_{15}$	Kaempferol 3-O-rutinoside	-	+	350	standard
39.60	939.10986	$C_{41}H_{32}O_{26}$	Penta-O-galloyl-hexose	+	+	270	nq
39.66	447.09321	$C_{21}H_{20}O_{11}$	Kaempferol 3-O-glucoside	-	+	350	standard
40.00	447.09368	$C_{21}H_{20}O_{11}$	Quercetin 3-O-rhamnoside	-	+	350	standard
44.30	1091.12172	$C_{48}H_{36}O_{30}$	Hexa-O-galloyl-hexose	-	+	270	nq
45.23	1091.12189	$C_{48}H_{36}O_{30}$	Hexa-O-galloyl-hexose	-	+	270	nq
45.86	431.09877	$C_{21}H_{20}O_{10}$	Kaempferol rhamnoside	-	+	350	Kaempferol 3-O-glucoside
48.51	1243.13256	$C_{55}H_{40}O_{34}$	Hepta-O-galloyl-hexose	-	+	270	nq
52.62	1395.14483	$C_{62}H_{44}O_{38}$	Octa-O-galloyl-hexose	-	+	270	nq

* for components for which standards were not available, compounds with similar spectral characteristics were used for quantification; nq – not quantified due to the lack of an appropriate standard, peak areas were used for model development

Table S2. Complete experimental design matrix and raw analytical responses obtained for *Hamamelis virginiana* leaves extracts.

Run	Temperature (°C); X1	MeOH/water (v/v); X2	Technique; X3	Number of cycles; X4	TPC (mg GAE/g DW)	DPPH (mg TRE/g DW)	Hamamelitannin (μg/g)	Ellagic acid (μg/g)	Sum flavonoids (μg/g)	Gallic acid derivatives (μg/g)	High-molecular-weight galloyl derivatives
1	60	50	ASE	Level 1 of E	45.3341	205.687	490.8	1093.17	2700.4	2593.78	3.16679E+08
2	40	40	ASE	Level 1 of E	43.6066	173.244	248.684	209.879	2353.29	2993.12	1.61447E+08
3	80	40	ASE	Level 3 of E	97.1694	275.393	632.092	1038.94	3229.08	4465.05	3.46105E+08†
4	55	65	ASE	Level 3 of E	102.368	297.187	438.384	2446.31	4072.52	3396.21	3.85081E+08
5	55	65	ASE	Level 3 of E	106.703	279.648	475.545	2676.7	3867.45	3488.54	4.08025E+08
6	55	65	ASE	Level 3 of E	114.944	298.335	465.805	2365.11	3733.99	3397.9	4.03757E+08
7	80	65	ASE	Level 1 of E	61.1935	249.114	411.482	1769.6	3050.54	2786.16	3.50583E+08
8	45	45	ASE	Level 2 of E	70.4549	230.693	412.927	458.498	2985.77	4048.62	2.44332E+08
9	40	45	ASE	Level 3 of E	88.1542	235.153	514.9	662.539	3168.89	3265.35	9.58206E+07†
10	75	95	ASE	Level 2 of E	81.6979	312.977	560.215	4096.12	4427.59	3538.91	3.49508E+08
11	40	80	ASE	Level 2 of E	77.8438	201.595	539.846	2619.92	3735.12	2910.16	2.26344E+08
12	55	100	ASE	Level 1 of E	42.6177	244.876	181.191	2797.04	3055.81	2303.91	3.53245E+08
13	40	100	ASE	Level 3 of E	73.8663	253.648	402.619	3385.97	3487.16	2672.58	2.8695E+08
14	80	100	ASE	Level 3 of E	88.8195	296.782	411.798	4597.04	4206.56	3212.51	2.74591E+08
15	65	40	UA	Level 1 of E	36.0557	219.445	292.073	607.072	2511.17	3110.23	2.81351E+08
16	80	100	UA	Level 1 of E	35.8911	206.716	227.038	2400.59	2598.95	2095.96	3.1556E+08
17	80	60	UA	Level 3 of E	105.316	358.465	314.195	2173.84	3517.51	3172.34	3.7312E+08
18	75	50	UA	Level 2 of E	70.8172	373.911	369.72	1830.14	3321.42	3462.23	4.32434E+08
19	40	40	UA	Level 3 of E	95.6303	251.585	254.575	237.868	2928.96	4248.59	1.99559E+08
20	40	75	UA	Level 1 of E	35.3411	259.517	335.668	2721.63	2800.54	2269.99	3.81446E+08
21	45	90	UA	Level 2 of E	70.7928	224.591	315.125	3030.3	3430.84	2748.6	2.08636E+08
22	45	70	UA	Level 3 of E	94.7438	276.791	355.907	3647.3	3581.43	3161.76	4.01825E+08
23	55	100	UA	Level 3 of E	87.9676	225.798	275.835	3503.69	3434.39	2682.26	4.22506E+08
24	60	50	UA	Level 3 of E	103.248	293.276	365.027	2910.96	3290.42	3330.83	3.95608E+08

Note: Highlighted cells indicate individual observations excluded from the regression model fitting based on diagnostic assessment performed using Design-Expert software. Potential outliers were evaluated using externally studentized residuals and Cook's distance to identify observations showing substantial deviations from model predictions or exerting a disproportionate influence on the fitted models. The excluded values are presented for transparency but were not included in the corresponding regression analyses.

Table S3. Complete experimental design matrix and raw analytical responses obtained for *Hamamelis virginiana* bark extracts.

Run	Temperature (°C); X1	MeOH/water (v/v); X2	Technique; X3	Number of cycles; X4	TPC (mg GAE/g DW)	DPPH (mg TRE/g DW)	Galloyl-hexose (mg/g)	Hamamelitannin (µg/g)	Gallic acid derivatives (mg/g)	High-molecular-weight galloyl derivatives**
1	60	50	ASE	Level 1 of E	38.6669	368.973	11.0668	55.3483	5.49622	6.649E+07
2	40	40	ASE	Level 1 of E	38.3751	333.506	10.9387	48.8367	5.67348	6.78581E+07
3	80	40	ASE	Level 3 of E	90.8379	386.932	13.3756	53.4927	7.43083	5.25608E+07
4	55	65	ASE	Level 3 of E	69.9875	316.604	11.1703	58.0316	5.53965	9.4518E+07
5	55	65	ASE	Level 3 of E	81.7245	364.979	11.6084	60.5283	5.52882	9.36577E+07
6	55	65	ASE	Level 3 of E	80.5815	403.044	10.3756	57.5901	5.2177	9.19992E+07
7	80	65	ASE	Level 1 of E	54.2833	376.373	10.9498	58.5352	6.62697	9.16859E+07
8	45	45	ASE	Level 2 of E	75.2994	453.593	11.234	56.6226	7.25597	9.13656E+07
9	40	45	ASE	Level 3 of E	81.3869	346.775	12.9748	50.3823	7.14815	8.35007E+07
10	75	95	ASE	Level 2 of E	51.9662	301.855	11.6542	61.0868	4.64421	1.01751E+08
11	40	80	ASE	Level 2 of E	50.0568	253.514†	11.3679	59.0876	6.81063	9.32508E+07
12	55	100	ASE	Level 1 of E	25.9698	215.26	6.99985	38.1859	3.31065	5.69373E+07
13	40	100	ASE	Level 3 of E	51.847	249.359	8.64863	49.5784	5.25342	7.62392E+07
14	80	100	ASE	Level 3 of E	60.3112	247.074	10.6509	57.0997	5.11789	8.94456E+07
15	65	40	UA	Level 1 of E	32.7808	331.354	8.99104	45.3452	5.28817	5.92095E+07
16	80	100	UA	Level 1 of E	22.6245	219.319	6.63476	38.0168	2.73267	4.87843E+07
17	80	60	UA	Level 3 of E	72.3952	380.738	12.2365	48.1077	5.91259	6.561E+07
18	75	50	UA	Level 2 of E	62.3366	391.367	10.2983	48.3082	6.37026	7.15887E+07
19	40	40	UA	Level 3 of E	72.4385	328.968	11.4772	47.0562	5.74331	5.97387E+07
20	40	75	UA	Level 1 of E	30.1301	282.246	8.96205	48.741	4.43319	6.86378E+07
21	45	90	UA	Level 2 of E	42.6031	309.769	9.09593	51.5853	3.33335	7.29915E+07
22	45	70	UA	Level 3 of E	64.427	324.462	10.0929	52.2226	5.27283	7.02351E+07
23	55	100	UA	Level 3 of E	69.7983	278.746	8.32284	49.2514	4.09605	6.59169E+07
24	60	50	UA	Level 3 of E	92.2469	344.161	10.7367	49.9346	5.95756	7.14175E+07

Note: Highlighted cells indicate individual observations excluded from the regression model fitting based on diagnostic assessment performed using Design-Expert software. Potential outliers were evaluated using externally studentized residuals and Cook's distance to identify observations showing substantial deviations from model predictions or exerting a disproportionate influence on the fitted models. The excluded values are presented for transparency but were not included in the corresponding regression analyses.

Table S4a. Analysis of variance (ANOVA) for the fitted response surface model of total phenolic content (TPC) in *Hamamelis virginiana* leaf extracts.

Source	Sum of Squares	df	Mean Square	F-value	p-value	
**Model**	14403.10	12	1200.26	44.37	< 0.0001	significant
A-Temp.	289.77	1	289.77	10.71	0.0074	
B-MeOH	134.06	1	134.06	4.96	0.0479	
C-Technique	120.71	1	120.71	4.46	0.0583	
D-Number of cycles	12151.12	2	6075.56	224.58	< 0.0001	
AB	42.16	1	42.16	1.56	0.2378	
BD	162.55	2	81.28	3.00	0.0910	
CD	140.82	2	70.41	2.60	0.1187	
A^2	84.17	1	84.17	3.11	0.1055	
B^2	550.58	1	550.58	20.35	0.0009	
**Residual**	297.58	11	27.05			
Lack of Fit	215.96	9	24.00	0.5880	0.7637	not significant
Pure Error	81.62	2	40.81			
**Cor Total**	14700.68	23				

Table S4b. Analysis of variance (ANOVA) for the fitted response surface model of DPPH in *Hamamelis virginiana* leaf extracts.

Source	Sum of Squares	df	Mean Square	F-value	p-value	
**Model**	52481.73	11	4771.07	29.47	< 0.0001	significant
A-Temp.	12426.24	1	12426.24	76.76	< 0.0001	
B-MeOH	46.44	1	46.44	0.2869	0.6020	
C-Technique	492.19	1	492.19	3.04	0.1068	
D-Number of cycles	11549.05	2	5774.53	35.67	< 0.0001	
AD	8361.77	2	4180.89	25.83	< 0.0001	
BC	3338.34	1	3338.34	20.62	0.0007	
BD	2896.09	2	1448.05	8.94	0.0042	
B^2	7288.12	1	7288.12	45.02	< 0.0001	
**Residual**	1942.71	12	161.89			
Lack of Fit	1723.32	10	172.33	1.57	0.4507	not significant
Pure Error	219.39	2	109.70			
**Cor Total**	54424.44	23				

Table S4c. Analysis of variance (ANOVA) for the fitted response surface model of hamamelitannin in *Hamamelis virginiana* leaf extracts.

Source	Sum of Squares	df	Mean Square	F-value	p-value	
**Model**	2.438E+05	10	24384.06	5.62	0.0025	significant
A-Temp.	15535.99	1	15535.99	3.58	0.0809	
B-MeOH	24548.05	1	24548.05	5.66	0.0334	
C-Technique	1.068E+05	1	1.068E+05	24.63	0.0003	
D-Number of cycles	50182.66	2	25091.33	5.79	0.0160	
AB	10699.64	1	10699.64	2.47	0.1403	
AC	12853.56	1	12853.56	2.96	0.1088	
CD	28777.56	2	14388.78	3.32	0.0685	
B^2	8221.27	1	8221.27	1.90	0.1918	
**Residual**	56379.73	13	4336.90			
Lack of Fit	55637.19	11	5057.93	13.62	0.0703	not significant
Pure Error	742.54	2	371.27			
**Cor Total**	3.002E+05	23				

Table S4d. Analysis of variance (ANOVA) for the fitted response surface model of ellagic acid in *Hamamelis virginiana* leaf extracts.

Source	Sum of Squares	df	Mean Square	F-value	p-value	
**Model**	3.123E+07	7	4.461E+06	16.60	< 0.0001	significant
A-Temp.	5.365E+05	1	5.365E+05	2.00	0.1769	
B-MeOH	2.362E+07	1	2.362E+07	87.90	< 0.0001	
C-Technique	1.641E+05	1	1.641E+05	0.6107	0.4459	
D-Number of cycles	2.295E+06	2	1.147E+06	4.27	0.0327	
BC	7.227E+05	1	7.227E+05	2.69	0.1205	
B^2	2.188E+06	1	2.188E+06	8.14	0.0115	
**Residual**	4.300E+06	16	2.688E+05			
Lack of Fit	4.248E+06	14	3.034E+05	11.61	0.0820	not significant
Pure Error	52253.86	2	26126.93			
**Cor Total**	3.553E+07	23				

Table S4e. Analysis of variance (ANOVA) for the fitted response surface model of sum flavonoids in *Hamamelis virginiana* leaf extracts.

Source	Sum of Squares	df	Mean Square	F-value	p-value	
**Model**	6.350E+06	11	5.772E+05	29.68	< 0.0001	significant
A-Temp.	3.240E+05	1	3.240E+05	16.66	0.0015	
B-MeOH	9.977E+05	1	9.977E+05	51.30	< 0.0001	
C-Technique	3.976E+05	1	3.976E+05	20.44	0.0007	
D-Number of cycles	2.806E+06	2	1.403E+06	72.14	< 0.0001	
AB	83977.62	1	83977.62	4.32	0.0598	
BC	1.952E+05	1	1.952E+05	10.03	0.0081	
BD	1.700E+05	2	84996.74	4.37	0.0375	
A^2	62299.32	1	62299.32	3.20	0.0987	
B^2	5.408E+05	1	5.408E+05	27.81	0.0002	
**Residual**	2.334E+05	12	19447.82			
Lack of Fit	1.752E+05	10	17521.89	0.6026	0.7614	not significant
Pure Error	58154.95	2	29077.47			
**Cor Total**	6.583E+06	23				

Table S4f. Analysis of variance (ANOVA) for the fitted response surface model of gallic acid derivatives in *Hamamelis virginiana* leaf extracts.

Source	Sum of Squares	df	Mean Square	F-value	p-value	
**Model**	7.304E+06	7	1.043E+06	23.57	< 0.0001	significant
A-Temp.	2.845E+05	1	2.845E+05	6.43	0.0221	
B-MeOH	3.359E+06	1	3.359E+06	75.89	< 0.0001	
C-Technique	2.292E+05	1	2.292E+05	5.18	0.0370	
D-Number of cycles	3.442E+06	2	1.721E+06	38.88	< 0.0001	
AC	5.474E+05	1	5.474E+05	12.37	0.0029	
B^2	3.909E+05	1	3.909E+05	8.83	0.0090	
**Residual**	7.083E+05	16	44268.74			
Lack of Fit	7.027E+05	14	50194.18	17.99	0.0539	not significant
Pure Error	5581.26	2	2790.63			
**Cor Total**	8.012E+06	23				

Table S4g. Analysis of variance (ANOVA) for the fitted response surface model of high-molecular-weight galloyl derivatives in *Hamamelis virginiana* leaf extracts.

Source	Sum of Squares	df	Mean Square	F-value	p-value	
**Model**	1.322E+17	13	1.017E+16	35.30	< 0.0001	significant
A-Temp.	1.359E+16	1	1.359E+16	47.18	0.0001	
B-MeOH	3.877E+15	1	3.877E+15	13.46	0.0063	
C-Technique	5.304E+15	1	5.304E+15	18.42	0.0026	
D-Number of cycles	6.253E+15	2	3.126E+15	10.85	0.0053	
AD	2.168E+16	2	1.084E+16	37.63	< 0.0001	
BD	1.656E+16	2	8.280E+15	28.75	0.0002	
CD	3.458E+15	2	1.729E+15	6.00	0.0256	
A^2	2.214E+16	1	2.214E+16	76.88	< 0.0001	
B^2	4.231E+16	1	4.231E+16	146.89	< 0.0001	
**Residual**	2.304E+15	8	2.880E+14			
Lack of Fit	2.006E+15	6	3.344E+14	2.25	0.3398	not significant
Pure Error	2.978E+14	2	1.489E+14			
**Cor Total**	1.345E+17	21				

Table S5a. Analysis of variance (ANOVA) for the fitted response surface model of total phenolic content (TPC) in *Hamamelis virginiana* bark extracts.

Source	Sum of Squares	df	Mean Square	F-value	p-value	
**Model**	8807.92	6	1467.99	26.41	< 0.0001	significant
A-Temp.	241.85	1	241.85	4.35	0.0524	
B-MeOH	1593.87	1	1593.87	28.67	< 0.0001	
C-Technique	134.87	1	134.87	2.43	0.1377	
D-Number of cycles	7031.90	2	3515.95	63.25	< 0.0001	
BC	126.13	1	126.13	2.27	0.1503	
**Residual**	945.02	17	55.59			
Lack of Fit	861.26	15	57.42	1.37	0.5015	not significant
Pure Error	83.77	2	41.88			
**Cor Total**	9752.95	23				

Table S5b. Analysis of variance (ANOVA) for the fitted response surface model of DPPH in *Hamamelis virginiana* bark extracts.

Source	Sum of Squares	df	Mean Square	F-value	p-value	
**Model**	69416.81	6	11569.47	16.49	< 0.0001	significant
A-Temp.	1477.33	1	1477.33	2.11	0.1661	
B-MeOH	50461.52	1	50461.52	71.91	< 0.0001	
C-Technique	2285.98	1	2285.98	3.26	0.0899	
D-Number of cycles	10377.56	2	5188.78	7.39	0.0053	
B^2	7512.30	1	7512.30	10.71	0.0048	
**Residual**	11227.81	16	701.74			
Lack of Fit	7474.13	14	533.87	0.2844	0.9421	not significant
Pure Error	3753.68	2	1876.84			
**Cor Total**	80644.62	22				

Table S5c. Analysis of variance (ANOVA) for the fitted response surface model of galloyl-hexose in *Hamamelis virginiana* bark extracts.

Source	Sum of Squares	df	Mean Square	F-value	p-value	
**Model**	60.87	9	6.76	20.60	< 0.0001	significant
A-Temp.	2.75	1	2.75	8.37	0.0118	
B-MeOH	27.72	1	27.72	84.43	< 0.0001	
C-Technique	8.67	1	8.67	26.41	0.0002	
D-Number of cycles	13.55	2	6.77	20.63	< 0.0001	
BD	4.12	2	2.06	6.28	0.0113	
A^2	3.66	1	3.66	11.16	0.0049	
B^2	1.75	1	1.75	5.32	0.0369	
**Residual**	4.60	14	0.3283			
Lack of Fit	3.81	12	0.3179	0.8139	0.6730	not significant
Pure Error	0.7811	2	0.3906			
**Cor Total**	65.46	23				

Table S5d. Analysis of variance (ANOVA) for the fitted response surface model of hamamelitannin in *Hamamelis virginiana* bark extracts.

Source	Sum of Squares	df	Mean Square	F-value	p-value	
**Model**	876.59	13	67.43	28.03	< 0.0001	significant
A-Temp.	27.68	1	27.68	11.51	0.0069	
B-MeOH	27.65	1	27.65	11.49	0.0069	
C-Technique	209.07	1	209.07	86.90	< 0.0001	
D-Number of cycles	157.71	2	78.85	32.78	< 0.0001	
AB	6.11	1	6.11	2.54	0.1422	
AC	68.50	1	68.50	28.47	0.0003	
BC	7.13	1	7.13	2.96	0.1160	
BD	116.69	2	58.35	24.25	0.0001	
CD	18.44	2	9.22	3.83	0.0581	
B^2	172.12	1	172.12	71.54	< 0.0001	
**Residual**	24.06	10	2.41			
Lack of Fit	19.04	8	2.38	0.9479	0.6079	not significant
Pure Error	5.02	2	2.51			
**Cor Total**	900.65	23				

Table S5e. Analysis of variance (ANOVA) for the fitted response surface model of gallic acid derivatives in *Hamamelis virginiana* bark extracts.

Source	Sum of Squares	df	Mean Square	F-value	p-value	
**Model**	29.96	7	4.28	13.64	< 0.0001	significant
A-Temp.	0.3160	1	0.3160	1.01	0.3305	
B-MeOH	18.81	1	18.81	59.94	< 0.0001	
C-Technique	4.94	1	4.94	15.73	0.0011	
D-Number of cycles	3.81	2	1.90	6.07	0.0109	
AB	1.06	1	1.06	3.37	0.0849	
A^2	2.49	1	2.49	7.94	0.0124	
**Residual**	5.02	16	0.3137			
Lack of Fit	4.95	14	0.3538	10.58	0.0896	not significant
Pure Error	0.0669	2	0.0334			
**Cor Total**	34.98	23				

Table S5f. Analysis of variance (ANOVA) for the fitted response surface model of high-molecular-weight galloyl derivatives in *Hamamelis virginiana* bark extracts.

Source	Sum of Squares	df	Mean Square	F-value	p-value	
**Model**	4.743E+15	10	4.743E+14	11.49	< 0.0001	significant
A-Temp.	1.462E+09	1	1.462E+09	0.0000	0.9953	
B-MeOH	3.117E+13	1	3.117E+13	0.7553	0.4006	
C-Technique	1.254E+15	1	1.254E+15	30.40	< 0.0001	
D-Number of cycles	8.273E+14	2	4.137E+14	10.02	0.0023	
AB	5.303E+14	1	5.303E+14	12.85	0.0033	
BC	9.306E+13	1	9.306E+13	2.26	0.1571	
BD	3.440E+14	2	1.720E+14	4.17	0.0399	
B^2	1.424E+15	1	1.424E+15	34.52	< 0.0001	
**Residual**	5.365E+14	13	4.127E+13			
Lack of Fit	5.332E+14	11	4.847E+13	29.57	0.0332	significant
Pure Error	3.279E+12	2	1.639E+12			
**Cor Total**	5.279E+15	23				

Table S6. Optimization criteria applied in the desirability function approach for the multi-response optimization of phenolic compound extraction from *Hamamelis virginiana* leaves.

Name	Goal	Lower Limit	Upper Limit	Lower Weight	Upper Weight	Importance
A:Temp.	is in range	40	80	1	1	3
B:MeOH	is in range	40	100	1	1	3
C:Technique	is in range	ASE	UA	1	1	3
D:Number of cylces	is in range	Level 1 of E	Level 3 of E	1	1	3
TPC (mg GAE/g DW)	maximize	35.3411	114.944	1	1	3
DPPH (mg TRE/g DW)	maximize	173.244	373.911	1	1	3
Hamamelitannin (µg/g)	maximize	181.191	632.092	1	1	3
Ellagic acid (µg/g)	maximize	238.742	689.191	1	1	3
Sum flavonoids (µg/g)	maximize	2353.29	4427.59	1	1	3
Gallic acid derivatives (µg/g)	maximize	94.5078	10048.6	1	1	3
High-molecular-weight galloyl derivatives	maximize	1.61447E+08	4.32434E+08	1	1	3

Table S7. Optimization criteria applied in the desirability function approach for the multi-response optimization of phenolic compound extraction from *Hamamelis virginiana* bark.

Name	Goal	Lower Limit	Upper Limit	Lower Weight	Upper Weight	Importance
A:Temp.	is in range	40	80	1	1	3
B:MeOH	is in range	40	100	1	1	3
C:Technique	is in range	ASE	UA	1	1	3
D:Number of cycles	is in range	Level 1 of E	Level 3 of E	1	1	3
TPC (mg GAE/g DW)	maximize	22.6245	92.2469	1	1	3
DPPH (mg TRE/g DW)	maximize	215.26	453.593	1	1	3
Galloyl-hexose (mg/g)	maximize	6.63476	13.3756	1	1	3
Hamamelitannin (µg/g)	maximize	38.0168	61.0868	1	1	3
Gallic acid derivatives (mg/g)	maximize	2.73267	7.43083	1	1	3
High-molecular-weight galloyl derivatives	maximize	4.87843E+07	1.01751E+08	1	1	3

Table S8. Calibration curve parameters for the analyzed compounds

Component	**Concentration range**	**Equation**	**Correlation (r^2)**
Gallic acid	10–100 µg/mL	31.3x – 9.65	0.9996
Hamamelitannin	20–200 µg/mL	1016x + 12.6	0.9998
Ellagic acid	5–50 µg/mL	57.4x – 41.03	0.9985
Myricetin 3-O-rhamnoside	10–50 µg/mL	32.4x – 5.13	0.9998
Quercetin 3-O-galactoside	1.0–5.0 µg/mL	33.2x – 5.16	0.9995
Quercetin-3-O-glucuronide	1.0–10.0 µg/mL	13.7x – 3.41	0.9998
Quercetin 3-O-glucoside	1.0–5.0 µg/mL	54.7x – 0.57	0.9995
Kaempferol 3-O-galactoside	1.0–5.0 µg/mL	16.9x – 3.09	0.9996
Kaempferol 3-O-rutinoside	0.5–5.0 µg/mL	11.4x – 0.99	0.9996
Kaempferol 3-O-glucoside	1.0–10.0 µg/mL	18.1x –1.22	0.9997
Quercetin 3-O-rhamnoside	5.0–25 µg/mL	21.1x – 3.22	0.9998

The RSD for each calibration point was below 0.5%

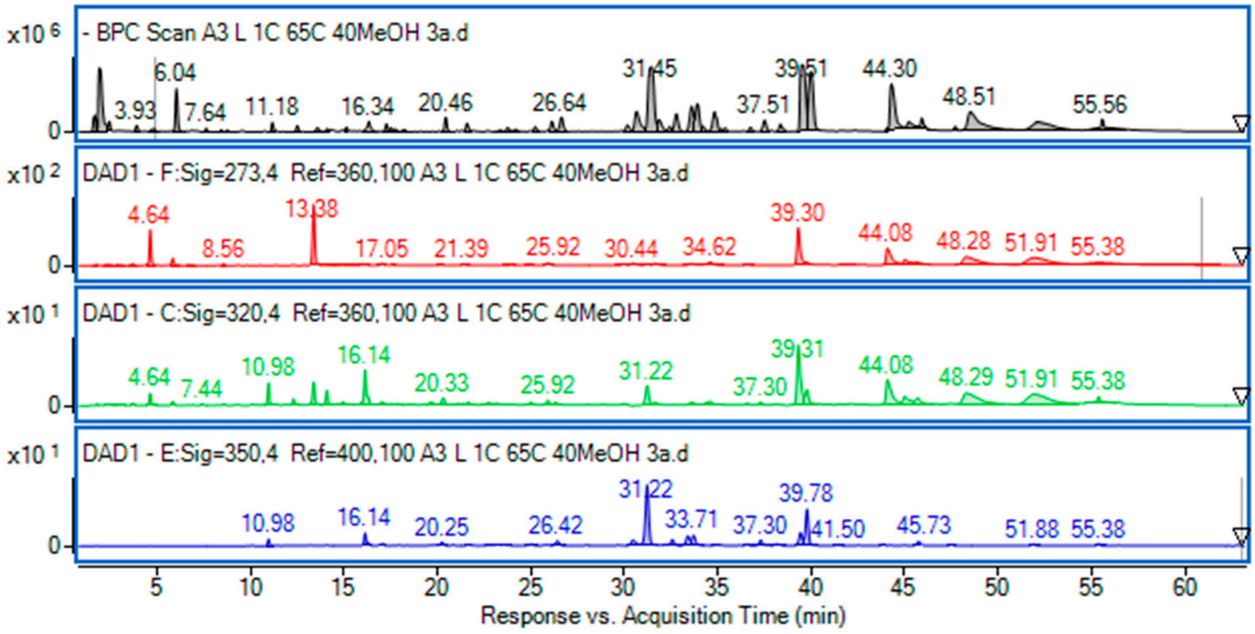

(a)

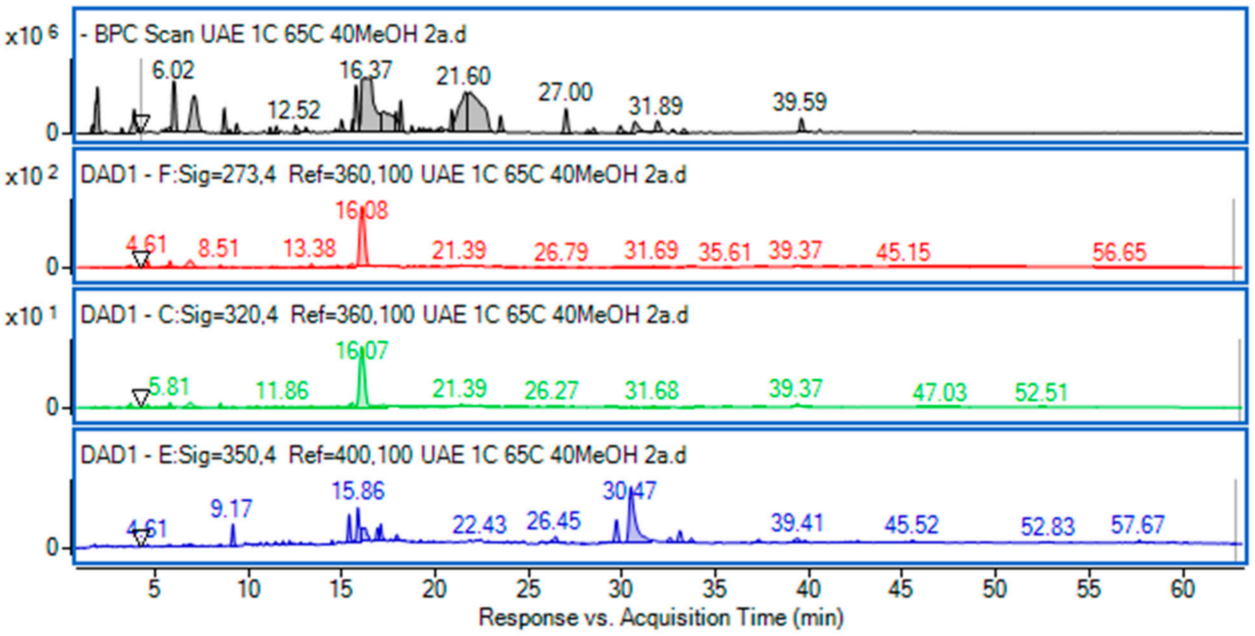

(b)

Figure S1. Representative chromatograms of *Hamamelis virginiana* leaf (a) and bark (b) extracts. Grey line – base peak chromatogram (BPC); red, green, and blue lines – DAD chromatograms recorded at 273, 320, and 350 nm, respectively.

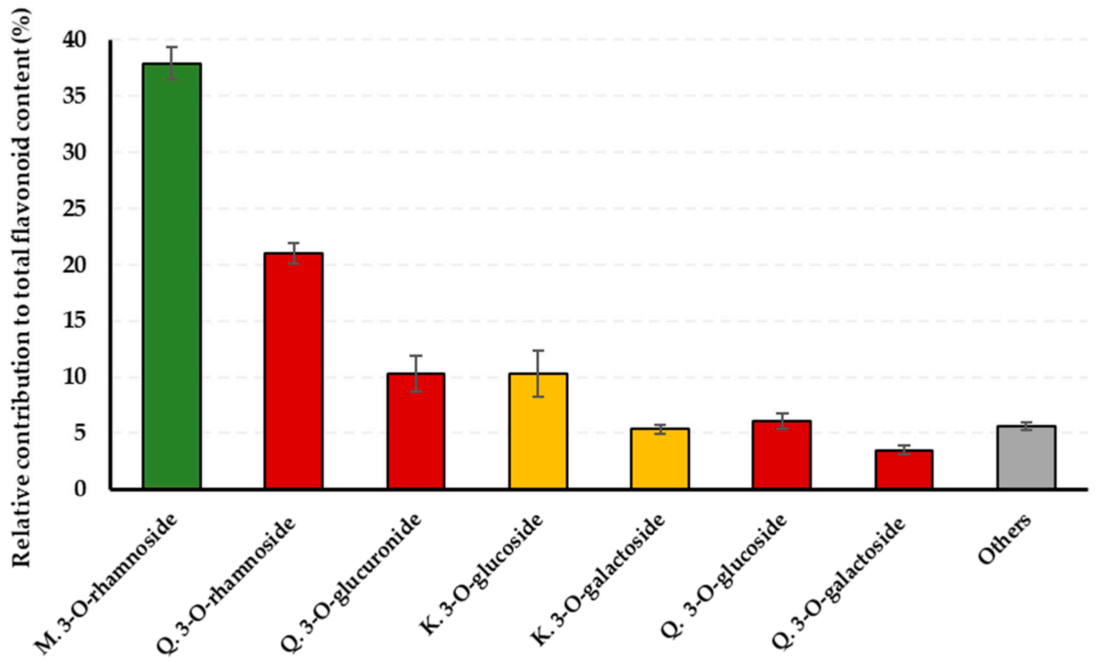

Figure S2. Relative contribution of individual flavonoids to the total quantified flavonoid content of *H. virginiana* leaf extracts. Values represent the mean ± SD calculated across the 22 experimental runs. M, myricetin; Q, quercetin; K, kaempferol.

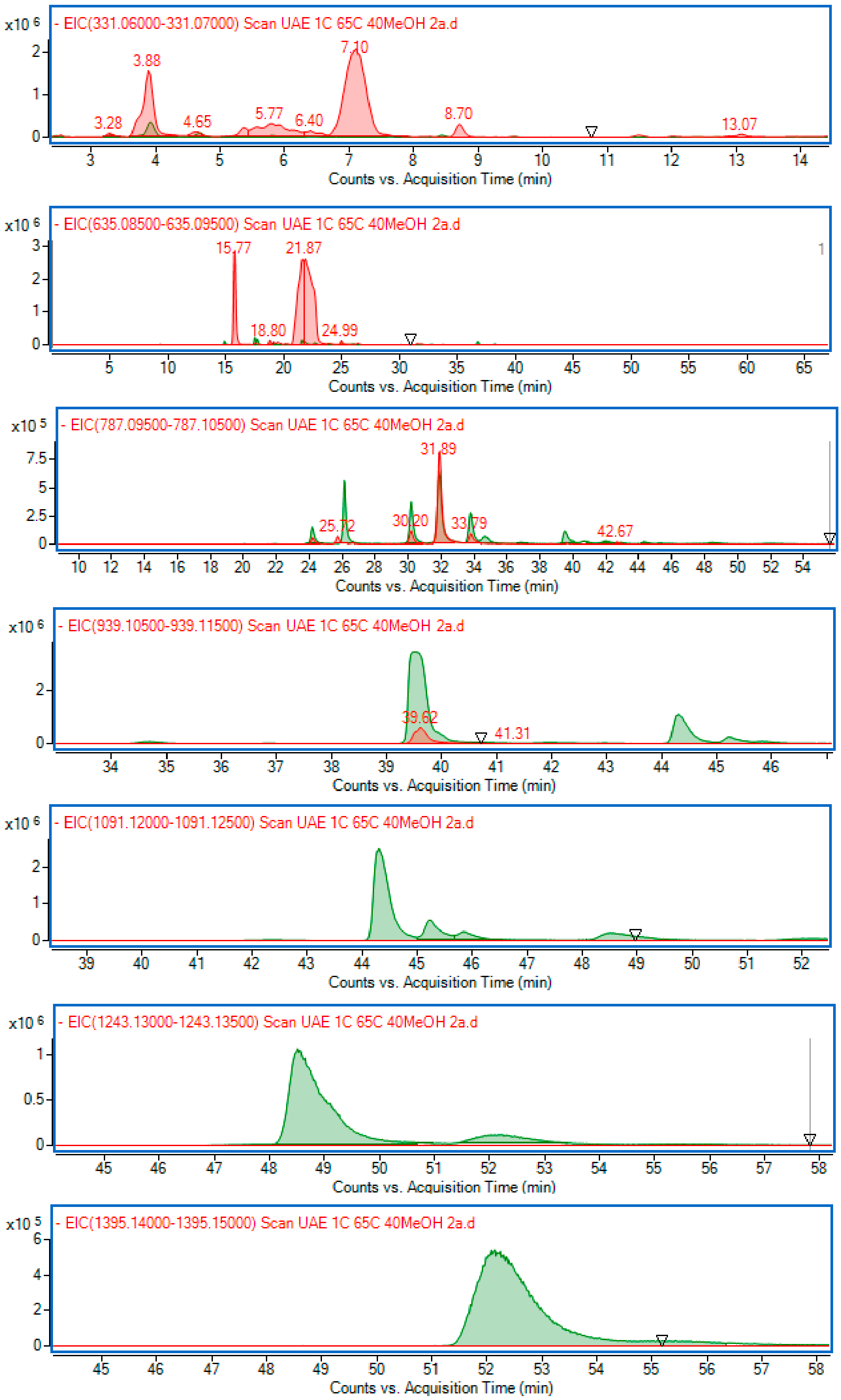

Figure S3. Extracted ion chromatograms (EICs) in the m/z ranges corresponding to galloylhexoses and high-molecular-weight tannins in H. virginiana leaf (green line) and bark (red line) extracts.

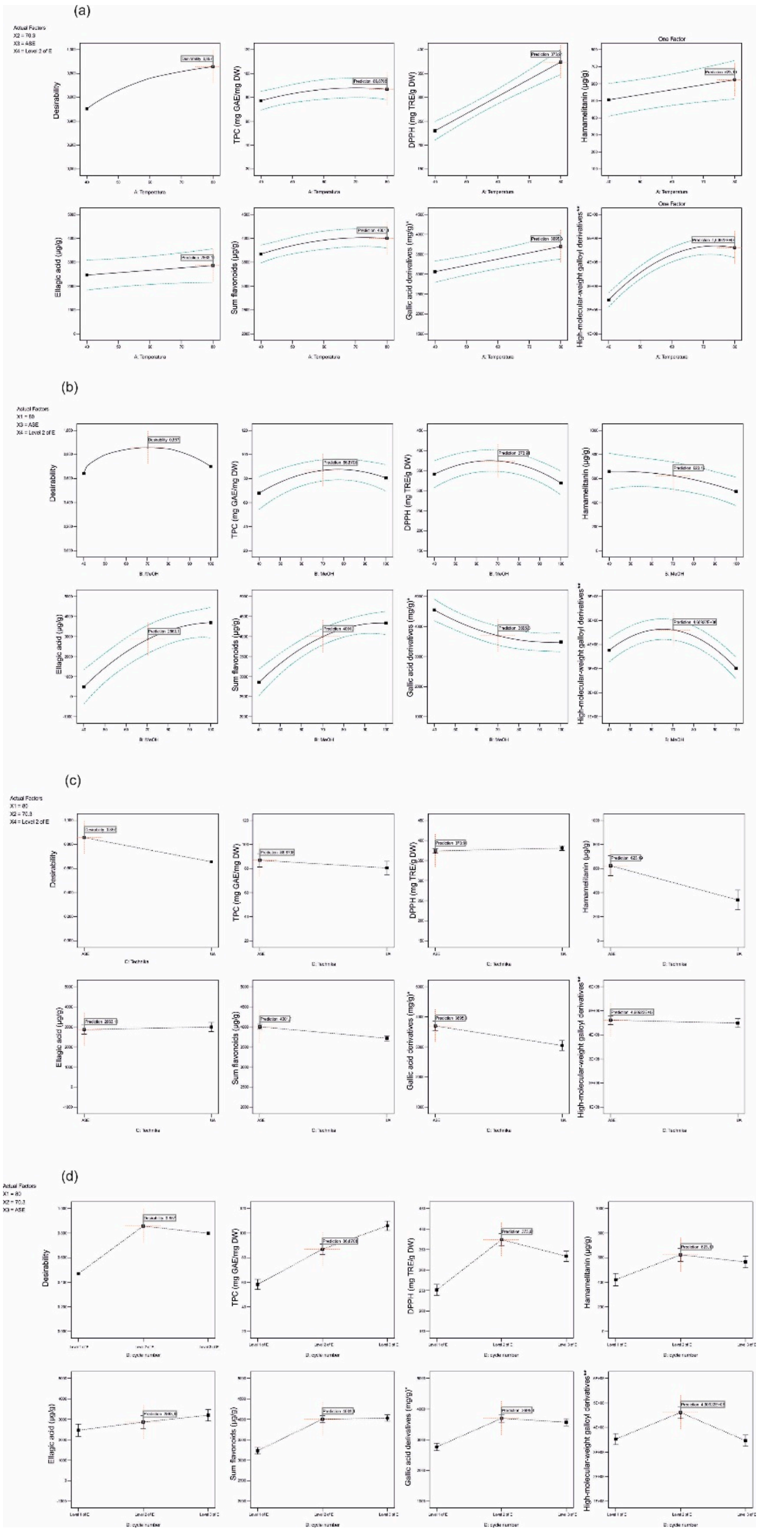

Figure S4. One-factor plots illustrating the effect of individual extraction factors on the predicted responses and overall desirability for leaf extracts: (a) extraction temperature, (b) methanol concentration, (c) extraction technique, and (d) number of extraction cycles, while the remaining factors were fixed at their respective optimal levels. The plots present the model-predicted effects on overall desirability, total phenolic content (TPC), DPPH radical-scavenging activity, hamamelitannin, ellagic acid, sum of flavonoids, gallic acid derivatives, and high-molecular-weight galloyl derivatives. The black lines represent predicted values, dashed green lines indicate confidence bands, and red dashed lines mark the factor settings corresponding to the optimum conditions.

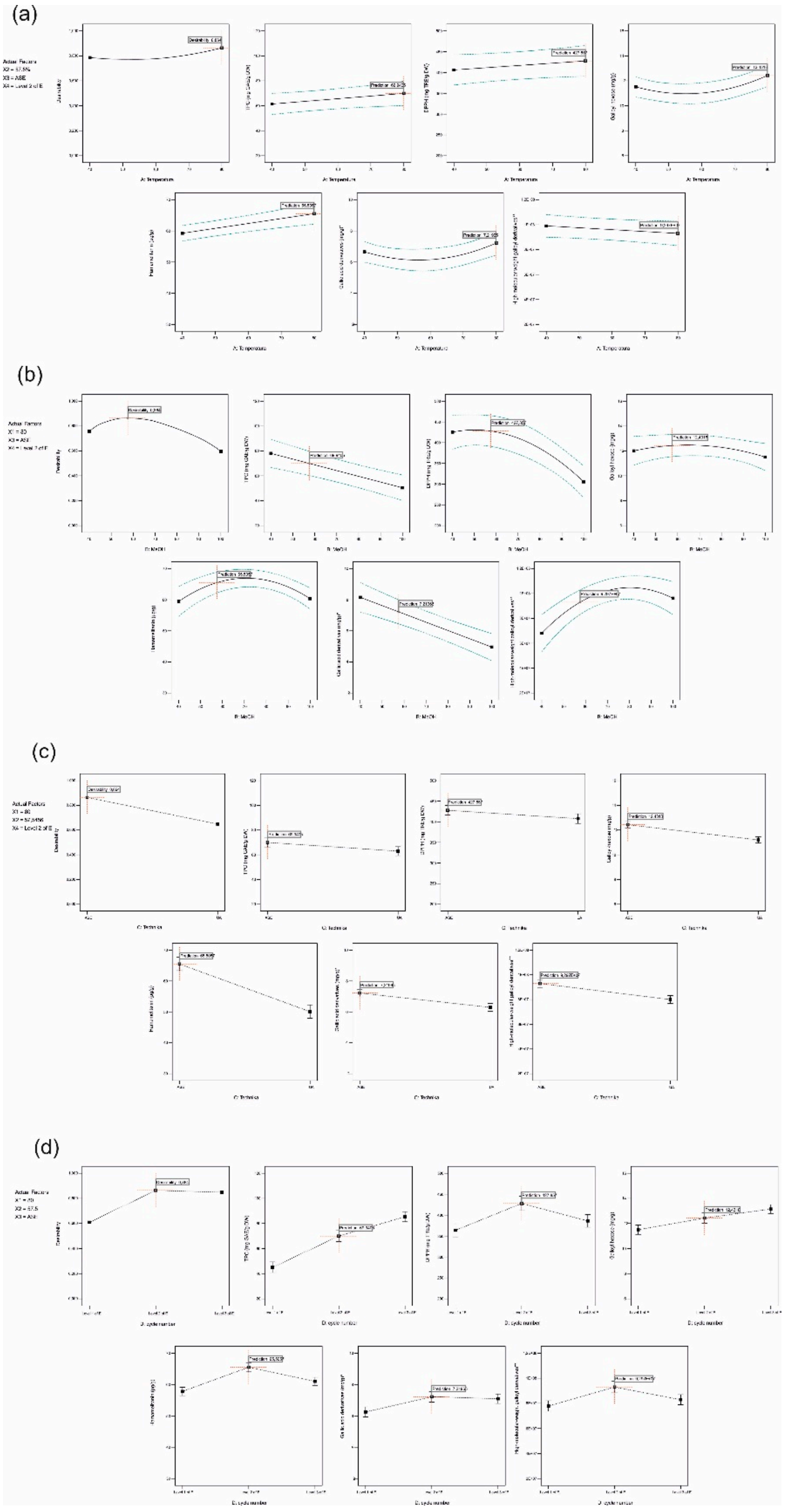

Figure S5. One-factor plots illustrating the effect of individual extraction factors on the predicted responses and overall desirability for bark extracts: (a) extraction temperature, (b) methanol concentration, (c) extraction technique (ASE or UAE), and (d) number of extraction cycles, while the remaining factors were fixed at their respective optimal levels. The plots show the model-predicted effects on overall desirability, total phenolic content (TPC), DPPH radical-scavenging activity, galloyl-hexose, hamamelitannin, gallic acid derivatives, and high-molecular-weight galloyl derivatives. Black lines and symbols represent model-predicted values, green dashed lines indicate the corresponding confidence limits, and red dashed lines indicate the factor settings selected under the optimal extraction conditions. For categorical and discrete factors, the plotted points represent predicted responses at the respective factor levels.
